# Mortality and morbidity in populations in the vicinity of coal mining: a systematic review

**DOI:** 10.1186/s12889-018-5505-7

**Published:** 2018-06-11

**Authors:** Javier Cortes-Ramirez, Suchithra Naish, Peter D Sly, Paul Jagals

**Affiliations:** 1 Child Health Research Centre, Faculty of Medicine, The University of Queensland, Brisbane, QLD Australia; 2 School of Public Health and Social Work, Faculty of Health, Queensland University of Technology, Brisbane, QLD Australia

**Keywords:** Mortality, Morbidity, Coal mining, Systematic review, International classification of diseases, Environmental health, Ecological studies, General population

## Abstract

**Background:**

Evidence of the association of coal mining with health outcomes such as increased mortality and morbidity in the general population has been provided by epidemiological studies in the last 25 years. Given the diverse sources of data included to investigate different health outcomes in the exposed populations, the International Classification of Diseases (ICD) can be used as a single classification standard to compare the findings of studies conducted in different socioeconomic and geographic contexts. The ICD classifies diagnoses of diseases and other disorders as codes organized by categories and chapters.

**Objectives:**

Identify the ICD codes found in studies of morbidity and/or mortality in populations resident or in proximity of coal mining and assess the methods of these studies conducting a systematic review.

**Methods:**

A systematic database search of PubMed, EMBASE and Scopus following the PRISMA protocol was conducted to assess epidemiological studies from 1990 to 2016. The health outcomes were mapped to ICD codes and classified by studies of morbidity and/or mortality, and the categories and chapters of the ICD.

**Results:**

Twenty-eight epidemiological studies with ecological design from the USA, Europe and China were included. The exposed populations had increased risk of mortality and/or morbidity by 78 ICD diagnosis categories and 9 groups of ICD categories in 10 chapters of the ICD: Neoplasms, diseases of the circulatory, respiratory and genitourinary systems, metabolic diseases, diseases of the eye and the skin, perinatal conditions, congenital and chromosomal abnormalities, and external causes of morbidity. Exposed populations had non-increased risk of 9 ICD diagnosis categories of diseases of the genitourinary system, and prostate cancer.

**Conclusions:**

There is consistent evidence of the association of coal mining with a wide spectrum of diseases in populations resident or in proximity of the mining activities. The methods of the studies included in this review can be integrated with individual-level and longitudinal studies to provide further evidence of the exposure pathways linked to increased risk in the exposed populations.

**Electronic supplementary material:**

The online version of this article (10.1186/s12889-018-5505-7) contains supplementary material, which is available to authorized users.

## Background

The impacts of coalmining on human health have been of scientific concern since the sixteen century [1]. Epidemiological research has provided evidence of the association of coal mining with diseases such as silicosis [2, 3] and chronic obstructive pulmonary disease [4] in studies of workers since the 1930s [5]. Epidemiological research has identified increased prevalence of chronic respiratory diseases [6], cardiovascular disease [7] and cancer [8], as well as physio-pathological mechanisms of respiratory diseases in coal miners [9]. The cumulated evidence of occupationally related studies is the main body of evidence about the association of diseases with exposure to coal mining.

In the last three decades epidemiological studies have increasingly investigated the impacts of coal mining on the general populations in proximity to coal mining [10]. These studies have found reduced health-related quality of life [11], increased perceptions of detrimental health conditions [12], and higher frequency of medical consultations [13] in communities resident or in proximity of coal mining. Research about the state of health of exposed populations has been conducted using data from self-report health status and health surveys [14, 15] or health care costs [16]. Studies using data from hospital records of these populations have found higher rates of morbidity and mortality due to respiratory diseases [10] and cancer [17], and measures of biomarkers have evidenced greater exposures to environmental contaminants associated with the mining activities [18–20]. Other studies have identified increased rates of dental disorders in coal mining regions of the USA [21] and Europe [22], and studies in mining regions of developing countries reported higher prevalence of parasitic diseases in communities residing nearby coal mining [23, 24]. The diverse health outcomes included in these studies show that the health impacts of coal mining on general population have been assessed using different approaches and sources of data.

This diversity of data is a challenge for more accurately determining the health outcomes associated with coal mining in populations in proximity to coal mining. Whereas surveys are important tools to measure health status, the indicators measured might not be comparable between studies conducted in different geographic and socioeconomic contexts [25]. On the other hand the biological effect of the exposures cannot always be established; for instance increased levels of biomarkers can be measured in people without having any manifestation of disease [20]. However health outcomes that can be categorized according to a standard such as the International Classification of Diseases (ICD) are reliable given the underlying medical diagnosis process, consistent between different regional contexts. The ICD classifies diagnoses in categories, blocks of categories, and chapters according to the organ systems and clinical criteria [26]. Records with medical diagnoses such as death certificates and hospital admissions register health outcomes that can be categorized with the ICD.

This paper presents a systematic review of studies of morbidity and/or mortality in populations resident or in proximity of coal mining and the diagnoses identified according to the ICD. The objectives of the study were to conduct a systematic search of epidemiological studies; identify the health outcomes found in the selected studies as classified by the ICD, and assess the methods of these studies.

## Methods

This review was done following the PRISMA protocol [27]. The protocol can be accessed at http://www.crd.york.ac.uk/PROSPERO/; (record: CRD42016052555). PRISMA checklist shown in Additional file 1.

Systematic searches of studies reported in PubMed, EMBASE and Scopus between January 1990 and October 2016 were conducted. Keyword combinations of “coal mining”, “coal”, “prevalence”, “incidence”, “mortality”, “morbidity”, “health impact”, “health outcome”, “international classification of diseases”, “hospitalization”, “hospital discharge”, “hospital separation”, “disease” and “death” were used. Search strategies shown in additional file (see Additional file 2).

### Eligibility criteria

For inclusion studies had to: 1) be designed to search for association between coal mining and morbidity and/or mortality in populations in proximity to coal mining; 2) obtain data from hospital records, death certificates and/or clinical assessments with medical diagnosis, 3) be published in English. Studies whose subjects of study were exclusively miners or workers were excluded.

### Paper selection and retrieval

**Fig. 1:**
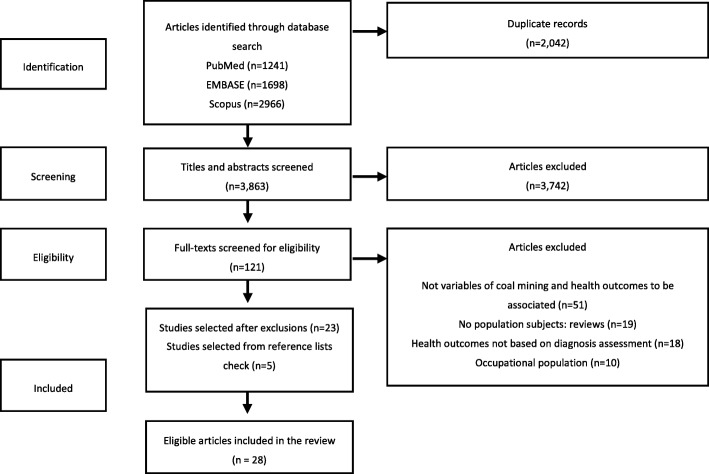
Studies selection and retrieval flowchart

Records were first identified in the search strategy, titles and abstracts were screened, and full-text reports selected and reviewed to identify eligible studies. A check of the reference lists of the reports selected for full-text review was done to identify potentially eligible studies (Fig. 1).

### Data extraction and synthesis

A data form pre-piloted with 10 studies was used to extract data from eligible studies. The studies were organized in three classes: studies of mortality, morbidity and, both mortality and morbidity, according to the sources of data (i.e. death certificates, hospital records and general practitioner consultations) (Data extraction form shown in Additional file 3).

The results of each study were assessed to identify measures of the association of coal mining with mortality and/or morbidity (i.e. risk measures: relative risk, odds ratio, rate ratio and regression coefficient) in exposed versus non-exposed populations. Exposed populations were defined as residents of coal mining areas (e.g. counties with coal mining within their boundaries) or populations in proximity to coal mining (e.g. towns < 5 km to coal mining). The health outcomes were matched with ICD codes of diagnosis categories, block of categories, and/or chapters according to the ICD-10 2016 revision Clinical Modification (ICD-10-CM) defined by the National Centre of Health Statistics [28]. An ICD was listed to have “increased risk”/“non-increased risk” if there were one or more increased/no increased significant risk measures for that ICD in the exposed populations. Significance was establish as measures with *p* < 0.05 and confidence intervals not crossing the null value. Significant covariates were extracted and classified as sociodemographic, smoking, obesity/overweight, environmental, and other comorbidities. ICDs reported with the ICD-9 revision and health outcomes not reported as ICDs were classified by a medical doctor.

Critical appraisal of the studies was done using a modified scale of the checklist proposed by Dufault and Klar [29]. The assessment was based on 10 items with a maximum overall score of 12 points (Table 1). For each item, if a study did not meet the criteria the item was given a score of “0”, otherwise it received a score of “1”, or “2”. The grade of the studies were labelled: *low* (< 5 points); *medium* (5-8 points) and *high relevance* (> 8 points).

**Table 1:** Assessment scale. Adapted from Dufault and Klar [29]

	Item	Description
Study design and focus (max = 4)	Sample size (max = 2)	Number of ecologic units included in the analysis as a proportion of the total number of units (3 levels: < 11% = 0 points; 11-79% = 1 point; > 79% = 2 points)
	Level of inference (max = 1)	The results of the analysis are not used to draw inferences for individuals
	Pre-specification of ecological units (max = 1)	Ecological units are selected to suit the hypothesis (as opposed to seemingly motivated by convenience or necessity such as the use of districts, towns or counties)
Statistical methodology (max = 5)	Validity of statistical inferences (max = 2)	Number of ecological units per covariate (3 levels: 0-10 = 0 points; 10-20 = 1 point; > 20 = 2 points)
	Use of covariates (max = 1)	Analysis adjusted for covariates (e.g. sociodemographic; environmental risk factors)
	Proper adjustment for covariates (max = 1)	Covariates are properly adjusted when regressed upon adjusted outcomes as recommended for ecological studies [30]
	Spatial effects (max = 1)	Inclusion of spatial analysis
Quality of reporting (max = 3)	Statement of study design (max = 1)	Key elements of the study design are presented in the report
	Justification of study design (max = 1)	Justification of the ecological analysis, the rationale and the objectives are presented in the report
	Discussion of cross-level bias and limitations (max = 1)	Readers are cautioned about the limitations of the ecological design and/or the ecological fallacy

Two independent researchers (JC and PJ) carried out the literature search, articles selection process, data extraction and synthesis between October 2016 and February 2017. Discrepancies were discussed with a third researcher (SN) until consensus was reached.

## Results

After exclusion of duplicates 3863 records were retrieved from the databases search. Initial titles and abstracts screening identified 121 potentially eligible studies. Independent full-text review reduced this number to 23. Five more studies were added from the reference lists for a final selection of 28 eligible studies (Fig. 1).

### Characteristics of the eligible studies

The 28 selected studies were published between October 2000 and April 2016. Twenty two (79%) were conducted in the USA in the region of Appalachia with the exception of one study in Illinois [30]. Three studies (11%) from China investigated populations in Shanxi province. Two studies (7%) from the UK were conducted in North East England. One study (4%) from Spain covered all Spanish towns in proximity to mining activities (Table 2).

**Table 2:** Characteristics of the studies selected in the review

[citation] authors (year)	Country	Study class	Health outcomes scoped	Analytical method	Critical appraisal (relevance)	Funding
[47] Liao et al. (2016)	China	Mortality/ Morbidity	Neural tube defects	Spatial autocorrelation	Medium	NNSFC-YTF-IGSNRS^a^
[31] Woolley et al. (2015)	USA	Mortality	Malignant and non-malignant respiratory diseases, and external and all causes of death	Two sample t test	Medium	ARIES ^b^
[49] Talbott et al. (2015)	USA	Morbidity	Diseases of the circulatory system	Linear regression, spatial regression	High	ARIES ^b^
[30] Mueller et al. (2015)	USA	Mortality/ Morbidity	Cancer of lung, colon, breast, prostate, and all combined cancer	Linear regression, spatial autocorrelation	High	CTMPHD-EFCR ^c^
[50] Lamm et al. (2015)	USA	Morbidity	Congenital anomalies	Poisson regression	Medium	ARIES ^b^
[32] Buchanich et al. (2014)	USA	Mortality	Malignant neoplasms, and external and all causes of death	Negative binomial regression	Medium	ARIES ^b^
[51] Brink et al. (2014)	USA	Morbidity	Diseases of the respiratory system	Linear regression	Medium	ARIES ^b^
[52] Liu et al. (2013)	USA	Morbidity	Diabetes mellitus	Multilevel linear regression	Medium	JHERC-NIOSH ^d^
[33] Fernandez-Navarro et al. (2012)	Spain	Mortality	Malignant neoplasms	Poisson regression, spatial regression	High	Spain Health Research Fund
[34] Borak et al. (2012)	USA	Mortality	All causes of death	Linear regression	Medium	National Mining Association
[35] Ahern and Hendryx (2012)	USA	Mortality	Malignant neoplasms	Linear regression	Medium	Not stated
[36] Esch and Hendryx (2011)	USA	Mortality	Chronic diseases of the circulatory system	Linear regression	High	Not stated
[53] Christian et al. (2011)	USA	Morbidity	Lung cancer	Spatial scan statistic	Medium	KLCRP ^e^
[37] Hendryx (2011)	USA	Mortality	All causes of death	Linear regression	Medium	Not stated
[54] Ahern et al. (2011)	USA	Morbidity	Congenital anomalies	Poisson regression, spatial autocorrelation	High	Not stated
[55] Ahern, et al. (2011)	USA	Morbidity	Low birth weight	Linear regression	Medium	Not stated
[46] Liao et al. (2010)	China	Mortality/Morbidity	Neural tube defects	Poisson regression, spatial autocorrelation	High	PNNSFC -HTRDPC –NBRPP ^f^
[38] Hitt and Hendryx (2010)	USA	Mortality	Cancer of the respiratory, digestive and genitourinary systems	Spatial autocorrelation	Medium	Not stated
[39] Hendryx et al. (2010)	USA	Mortality	Diseases of the respiratory and circulatory systems, all Malignant neoplasms, and all causes of death	Linear regression	Medium	Not stated
[40] Hendryx et al. (2010)	USA	Mortality	Cancer of the respiratory, digestive, genitourinary, hematopoietic, and central nervous systems, and melanoma	Linear regression, spatial autocorrelation	High	Not stated
[41] Hendryx and Ahern (2009)	USA	Mortality	All causes of death	Linear regression	Medium	Grant RRI-UWV ^g^
[42] Hendryx (2009)	USA	Mortality	Diseases of the circulatory, respiratory, and genitourinary systems	Poisson regression	Medium	Grant RRI-UWV
[43] Hendryx et al. (2008)	USA	Mortality	Lung cancer	Linear regression	Medium	Grant RRI-UWV
[44] Hendryx (2008)	USA	Mortality	All causes of death	Linear regression	Medium	Not stated
[56] Hendryx et al. (2007)	USA	Morbidity	Lung cancer, diseases of the respiratory, circulatory, and musculoskeletal systems, diabetes, and mental disorders	Multilevel logistic regression	Medium	Not stated
[48] Gu et al. (2007)	China	Mortality/ Morbidity	Neural tube defects	Spatial autocorrelation	Medium	Grant ^h^
[57] Howel et al. (2001)	UK	Morbidity	Diseases of the eyes and skin, and diseases of the respiratory system	Linear regression	Medium	Grant ^i^
[58] Pless-Mulloli et al. (2000)	UK	Morbidity	Diseases of the eyes and skin, and diseases of the respiratory system	Linear regression	Medium	Grant ^j^

^a^ National Natural Science Foundation of China and the Yong Talent Fund of the Institute of Geographic Sciences and the Natural Resources Search

^b^ Industrial affiliate program at Virginia Tech; supported by members that include companies in the energy sector

^c^ The Caryll Towsley Moy PhD Endowed Fund for Collaborative Research

^d^ The Johns Hopkins Education and Research Centre, National Institute of Occupational Safety and Health

^e^ Kentucky Lung Cancer Research Program

^f^ The Project of the National Natural Science Foundation of China, the Hi-Tech Research and Development Program of China, the National Basic Research Priorities Program of the Ministry of Science and Technology of the People’s Republic of China, and the Knowledge Innovation Program

^g^ Regional Research Institute, West Virginia University

^h^ National project on Population and Health, China

^i^ Medical Research Council (grant AIR/96/9) UK

^j^ Northern and Yorkshire Regional research and development grant UK

Fourteen studies (50%) retrieved data from death certificates (studies of mortality), 10 (36%) from hospital records or general practitioner consultations (studies of morbidity), and 4 (14%) from both death certificates and hospital records (studies of mortality and morbidity).

### Health outcomes and ICD codes

Thirteen (46%) of the eligible studies presented health outcomes already classified with the ICD-9 or ICD-10-CM revisions. Twenty three (82%) of the studies found significant risk measures for 88 ICD categories (single diagnosis), 9 blocks of ICD (groups of categories within the same chapter), 4 whole ICD chapters and 2 groups of combined chapters, in 10 out of 21 chapters of the ICD. These chapters include: neoplasms, diseases of the circulatory system, diseases of the respiratory system, diseases of the genitourinary system, endocrine and metabolic diseases, diseases of the eye and adnexa, diseases of the skin and subcutaneous tissue, conditions of the perinatal period, congenital malformations and chromosomal abnormalities, and external causes of morbidity. Five (18%) of the studies found non-significant risk measures (all increased) for ICD categories in the chapters: diseases of the circulatory system, congenital malformations and chromosomal abnormalities, and all combined chapters. Of all ICD categories, 31 were neoplasms, all malignant (i.e., cancer), affecting the following organ systems: integumentary, skeletal, endocrine, lymphatic and immune, respiratory, urinary, digestive, reproductive, and central nervous systems. Tables 3 and 4 show the list of ICD, organised by class of study and ICD chapter.

**Table 3:** ICD-10-CM diagnosis categories, block of categories and chapters identified in studies of mortality and mortality/morbidity

ICD	Increased risk y/n / NS^a^ / O^a^ [citation]	Total exposed	Values
Chapter: Neoplasms			
C00-C97 (All malignant neoplasms)	y [30, 32, 38–40]	Population in 214 USA counties	RR ranging 1.01-1.06 *p* < 0.05. Increased regression coefficients for residence and coal production ^c^
C15 (Cancer of oesophagus)	y [35]	Population in 82 USA counties	*r* = 0.766(SE = 0.353) *p* < 0.05 SMR in coal mining counties
C16 (Cancer of stomach)	y [35]	Population in 82 USA counties	*r* = 0.935(SE = 0.482) *p* < 0.05 SMR in coal mining counties
C18, C19, C20, C21 (Cancer of: colon, recto-sigmoid, rectum, anus)	y [30, 33, 35]	Population in 157 USA counties and 48 Spanish towns	RR = 1.27(1.12-1.44) MR men-women and RR = 1.31(1.13-1.52) MR men in towns <5Km to coal mining. Increased regression coefficients for residence, coal production and type of coal mining county ^c^
C22 (Cancer of liver and intrahepatic bile ducts)	y [33, 35]	Population in 82 USA counties and 48 Spanish towns	*r* = 0.788(SE = 0.395) *p* < 0.05 SMR in coal mining counties. RR = 1.69(1.09-2.63) MR men in towns <5Km to coal mining
C32 (Cancer of larynx)	y [40]	Population in 29 USA counties	r/adjusted R2 = 0.36/0.42 *p* < 0.002 SMR in coal mining counties
C33 (Cancer of trachea)	y [40]	Population in 29 USA counties	r/adjusted R2 = 0.36/0.42 *p* < 0.002 SMR in coal mining counties
C34 (Cancer of bronchus and lung)	y [30, 33, 35, 40, 43]	Population in 82 USA counties and 48 Spanish towns	RR = 1.22(1.01-1.49) MR men-women, RR = 1.29(1.05-1.59) MR men in towns <5Km to coal mining. Increased regression coefficients for residence, coal production, type of mining and type of coal mining county ^c^
C30-C39 (Cancer of respiratory and intrathoracic organs)	y [38] / O [31]	Population in 29 USA counties	Pearson coefficient = 0.47 *p* < 0.01 SMR in counties with 1000Tons mined /Km2. O: increased rates in a graphical analysis and comparative t-test
C43 (Melanoma)	y [40]	Population in 29 USA counties	r/adjusted R2 = 0.441/0.16 *p* < 0.002 SMR coal mining counties, *r* = 0.324/0.1 *p* < 0.02 SMR by coal production
C53 (Cervical cancer)	y [35]	Population in 82 USA counties	*r* = 0.699(SE = .325) *p* < 0.05 SMR in coal mining counties
C61 (Prostate cancer)	n [30]	Population in 75 USA counties	*r* = −0.32 *p* ≤ 0.005 SMR by coal production and type of coal mining county
C67 (Cancer of bladder)	y [35]	Population in 82 USA counties	*r* = 1.33(SE = .438) *p* < 0.01 SMR in coal mining counties
C70 (Cancer of meninges)	y [40]	Population in 29 USA counties	*r* = 0.441/0.16 *p* < 0.002 SMR in coal mining counties, *r* = 0.324/0.1 *p* < 0.02 SMR by coal production
C71 (Brain cancer)	y [33, 40]	Population in 29 USA counties and 48 Spanish towns	RR = 1.75(1.19-2.57) MR men in towns <5Km to coal mining. Increased regression coefficients for residence and type of mining ^c^
C72 (Cancer of spinal cord, cranial nerves and other parts of central nervous system)	y [40]	Population in 29 USA counties	*r/adjusted R2* = 0.441/0.16 *p* < 0.002 SMR in coal mining counties, *r* = 0.324/0.1 *p* < 0.02 SMR by coal production
C73 (Thyroid cancer)	y [33]	Population in 48 Spanish towns	RR = 2.05(1.01-4.13) MR men, RR = 1.70(1.02-2.84) MR women, in towns <5Km to coal mining
C81 (Hodgkin lymphoma)	y [40]	Population in 29 USA counties	*r/adjusted R2* = 0.441/0.16 *p* < 0.002 SMR in coal mining counties, *r* = 0.324/0.1 *p* < 0.02 SMR by coal production
C82, C83, C84, C85 (Follicular/non-follicular, mature T/NK-cell, and other specified/unspecified non-Hodgkin lymphomas	y [40]	Population in 29 USA counties	*r/adjusted R2* = 0.441/0.16 *p* < 0.002 SMR in coal mining counties, *r* = 0.324/0.1 *p* < 0.02 SMR by coal production
C88 (Malignant immuno-proliferative diseases and other B-cell lymphomas)	y [40]	Population in 29 USA counties	*r/adjusted R2* = 0.441/0.16 *p* < 0.002 SMR in coal mining counties, *r* = 0.324/0.1 *p* < 0.02 SMR by coal production
C90 (Multiple myeloma and plasmacytoma)	y [40]	Population in 29 USA counties	*r/adjusted R2* = 0.441/0.16 *p* < 0.002 SMR in coal mining counties, *r* = 0.324/0.1 *p* < 0.02 SMR by coal production
C91, C92, C93, C94, C95 (Lymphoid, myeloid, monocytic, other specified leukemia, unspecified leukemia)	y [35, 40]	Population in 82 USA counties	*r* = 1.102(SE = .554) *p* < 0.05 SMR in coal mining counties. r/adjustedR2 = 0.441/0.16 *p* < 0.002 SMR in coal mining counties, *r* = 0.324/0.1 *p* < 0.02 SMR by coal production
C96 (Unspecified cancer of lymphoid, hematopoietic and related tissue)	y [40]	Population in 29 USA counties	*r/adjusted R2* = 0.441/0.16 *p* < 0.002 SMR in coal mining counties, *r* = 0.324/0.1 *p* < 0.02 SMR by coal production
Chapter: Diseases of the circulatory system			
I00-I78 (All diseases of the circulatory system, except unclassified and unspecific)	y [39] / NS [39]	Population in 139 USA counties	*r* = 14.32(6.61) *p* < 0.03 SMR in high production coal mining counties. NS. 5.17 (5.97) *p* < 0.39 SMR in low production coal mining counties
I10 (Essential Hypertension)	y [36, 42] / n [42] ^b^	Population in 129 USA counties	RR ranging 0.96-1.28 in coal mining counties ^c^. *r* = 16.9(7.5) *p* < 0.03 SMR in coal mining counties, *r* = 11.4(5.5) *p* < 0.05 SMR by coal production
I11 (Hypertensive heart disease)	y [36, 42] / n [42] ^b^	Population in 129 USA counties	RR ranging 0.96-1.28 in coal mining counties ^c^. *r* = 16.9(7.5) *p* < 0.03 SMR in coal mining counties, *r* = 11.4(5.5) *p* < 0.05 SMR by coal production
I12 (Hypertensive chronic kidney disease)	y / n [42] ^b^	Population in 129 USA counties	RR ranging 0.96-1.28 in coal mining counties ^c^.
I13 (Hypertensive heart and chronic kidney disease)	y [36]	Population in 90 USA counties	*r* = 16.9(7.5) *p* < 0.03 SMR in coal mining counties, *r* = 11.4(5.5) *p* < 0.05 SMR by coal production
I21 (ST elevation and non-ST myocardial infarction)	y [36, 42] / n [42] ^b^	Population in 129 USA counties	RR ranging 0.96-1.28 in coal mining counties ^c^. *r* = 16.9(7.5) *p* < 0.03 SMR in coal mining counties, *r* = 11.4(5.5) *p* < 0.05 SMR by coal production
I24 (Other acute ischemic heart diseases)	y / n [42] ^b^	Population in 129 USA counties	RR ranging 0.96-1.2) in coal mining counties ^c^
I25 (Chronic ischemic heart disease)	y [36, 42] /n [42] ^b^	Population in 129 USA counties	RR ranging 0.96-1.2) in coal mining counties ^c^. *r* = 16.9(7.5) *p* < 0.03 SMR in coal mining counties, *r* = 11.4(5.5) *p* < 0.05 SMR by coal production
I31, I33, (Endocarditis, other diseases of pericardium)	y / n [42] ^b^	Population in 129 USA counties	RR ranging 0.89-1.10 in coal mining counties ^c^
I40 (Acute myocarditis)	y / n [42] ^b^	Population in 129 USA counties	RR ranging 0.89-1.10 in coal mining counties ^c^
I50 (Heart failure)	y [36, 42] /n [42] ^b^	Population in 129 USA counties	RR ranging 0.89-1.10 in coal mining counties ^c^. *r* = 16.9(7.5) *p* < 0.03 SMR in coal mining counties, *r* = 11.4(5.5) *p* < 0.05 SMR by coal production
I70 (Atherosclerosis)	y [36]	Population in 90 USA counties	*r* = 16.9(7.5) *p* < 0.03 SMR in coal mining counties, *r* = 11.4(5.5) *p* < 0.05 SMR by coal production
Chapter: Diseases of the respiratory system (J00-J99)	y [39] / O [31]	Population in 139 USA counties	*r* = 6.29(SE = 1.79) *p* < 0.001 SMR in coal mining counties, *r* = 9.81(SE = 2.32) *p* < 0.0001 SMR by type of coal mining county. O: increased rates in a graphical analysis and comparative t-test
J12, J13, J14, J15, J16, J17, J18 (Viral pneumonia, and pneumonia due to: streptococcus, Haemophilus influenzae, other bacteria, non-classified organism, unspecified organism)	y / n [42] ^b^	Population in 129 USA counties	RR ranging 0.89-1.13 in coal mining counties ^c^
J20, J21 (Acute bronchitis, bronchiolitis)	y / n [42] ^b^	Population in 129 USA counties	RR ranging 0.89-1.13 in coal mining counties ^c^
J22 (Unspecified acute lower respiratory infection)	y / n [42] ^b^	Population in 129 USA counties	RR ranging 0.89-1.13 in coal mining counties ^c^
J40, J41, J42 (Mucopurulent, simple, and not specified Bronchitis)	y / n [42] ^b^	Population in 129 USA counties	RR = 1.07(1.04-1.10) males, RR = 1.11(1.07-1.15) females; in coal mining counties
J43, J44 (Emphysema, other chronic obstructive pulmonary disease)	y / n [42] ^b^	Population in 129 USA counties	RR = 0.94(0.90-0.98) females in coal mining counties
J45 (Asthma)	y / n [42] ^b^	Population in 129 USA counties	RR = 1.04(1.02-1.06) males in coal mining counties
Chapter: Diseases of the genitourinary system			
N03, N04, N05 (Chronic/unspecified nephritic syndrome, nephrotic syndrome)	y [42]	Population in 129 USA counties	RR ranging 1.08-1.19 in coal mining counties ^c^
N17, N18, N19 (Acute and unspecific kidney failure, chronic kidney disease)	y [42]	Population in 129 USA counties	RR ranging 1.08-1.19 in coal mining counties ^c^
Chapter: External causes of morbidity (V00-V99)	y / n [32] ^b^ / O [31]	Population in 31 USA counties	RR ranging 0.91-1.12 in coal mining counties ^c^. O: increased rates in a graphical analysis and comparative t-test
ICD categories in more than one ICD chapter			
A00-Y89 (All combined internal and external causes)	y [32, 39, 41] / n [32] ^b^ / NS [34] / O [31]	Population in 139 USA counties	RR ranging 0.97-1.03 in coal mining counties ^c^. Increased regression coefficients for residence and type of mining ^c^. NS: *r* = 4.68(8.96) *p* < 0.6003 SMR in coal mining counties ^d^. O: increased rates in a graphical analysis and comparative t-test
A00-R99 (All combined internal causes)	y [37, 44]	Population in 139 USA counties	Adjusted *r* ranging 21.5-63.0 SMR by residence and type of mining ^c^
ICD categories in studies of mortality and morbidity			
Chapter: Congenital malformations, deformations and chromosomal abnormalities			
Q00, Q01, Q05, Q04.2 (Anencephaly, encephalocele, spina bifida, holoprosencephaly)	y [46] / O [47] / O-NS [48]	204 geographical grid points in Heshun province (China). 46 villages in Zhongyang and Jiaokou Counties (China)	RR = 1.338(1.004-1.783) *p* < 0.05 cases to mothers resident in coal mining areas. O: increased rate cases in populations < 8 km to coal transport roads (*p* < 0.007), not measure provided. O-NS: higher mortality rate (281.2/10000) in villages closer to coal mining plants/control population: chi-square test (*p* < 0.364)

Increased risk y/n: one or more risk measure increased/non-increased in exposed versus non-exposed populations. *r* regression coefficient, *SE* standard error, *MR* mortality rate, *RR* relative risk, *SMR* standardised mortality rate

^a^ NS/O: Not significant/Other measure

^b^ Disparities found in different exposed sub-groups

^c^ All risk measures provided in additional material [see Additional file 4].

^d^ These results were disputed, and the article subjected to erratum

**Table 4:** ICD-10-CM diagnosis categories, block of categories and chapters identified in studies of morbidity

ICD	Increased risk y/n / NS^a^ [citation]	Total exposed	Values
Chapter: Neoplasms			
C18-C21 (Colorectal cancer)	y [30]	Population in 75 USA counties	*r* = 0.37 *p* < 0.009 incidence rate in recently mined coal mining counties
C34 (Cancer of bronchus and lung)	y [30, 53]	Population in 75 USA counties	Adjusted *r* = 0.36 *p* < 0.001 incidence rate in coal mining counties. RR = 1.21 *p* < 0.01 and RR = 1.17 *p* < 0.01 in 2 clusters with coal mining counties
Chapter: Endocrine, nutritional and metabolic diseases			
E10, E11, E13 (Diabetes mellitus type 1 and 2, other specified diabetes mellitus)	y [52]	Population in 225 USA minor civil divisions	*r* = 0.116(0.059) *p* < 0.05 blood sugar levels by coal mining area density, *r* = 0.124(0.056) *p* < 0.01 blood sugar levels by proximity to abandoned coalmines
Chapter: Diseases of the eye and adnexa			
H57.9 (Unspecific disorder of eyes)	y / n [57, 58] ^b^	5 northern England communities (2000-20,000 persons)	Adjusted OR = 0.23(0.10-0.49) GP-consultations in 1 of 5 communities (1.4 km to coal mining). Adjusted OR = 1.43(1.20-1.70) GP-consultations in all other communities (0.8-1.3 Km to coal mining)
Chapter: Diseases of the circulatory system (I00-I99)	NS [49]	Population in 28 USA counties	Adjusted hospitalisation rates in coal mining counties by type and coal production = r ranging 0.01-0.05 (*p* > 0.05)
I10, I11, I12, I13, I15 (Primary HTA, hypertensive heart disease and chronic kidney disease, secondary hypertension)	y [56]	Population in 73 USA counties	OR = 1.003(1.001-1.005) hospitalisation rates in coal mining counties by coal production
Chapter: Diseases of the respiratory system (J00-J99)	y [51]	Population in 28 USA counties	*r* = 0.064(0.025) men-women, *r* = 0.064(0.022) *p* < 0.006 men, *r* = 0.063(0.029) *p* < 0.032 women; hospitalisation rates in coal mining counties, by coal production
J40, J41, J42, J43, J44, J47 (Specified/unspecified bronchitis, emphysema, chronic obstructive pulmonary disease, bronchiectasis)	y [56]	Population in 73 USA counties	OR = 1.003(1.001-1.006) hospitalisation rates in coal mining counties
J98.9 Unspecific respiratory disorders	y / n [57, 58] ^b^	5 northern England communities (2000-20,000 persons)	Adjusted OR = 0.23(0.10-0.49) GP-consultations in 1 of 5 communities (1.4 km to coal mining). Adjusted OR = 1.43(1.20-1.70) GP-consultations in all other communities (0.8-1.3 Km to coal mining)
Chapter: Diseases of the skin and subcutaneous tissue			
L98.9 (Unspecific disorders of skin)	y / n [57, 58] ^b^	5 northern England communities (2000-20,000 persons)	Adjusted OR = 0.23(0.10-0.49) GP-consultations in 1 of 5 communities (1.4 km to coal mining). Adjusted OR = 1.43(1.20-1.70) GP-consultations in all other communities (0.8-1.3 Km to coal mining)
Chapter: Diseases of the genitourinary system			
N00.3, N00.8, N00.9, N01.3, N02.2, N03, N04, N05, N08 (Acute nephritic syndromes, hematuria with diffuse membranous glomerulonephritis, chronic nephritic syndrome, nephrotic syndrome, unspecified nephritic syndrome and glomerular disorders classified elsewhere)	n [56]	Population in 73 USA counties	OR = 0.997(0.994-0.999) hospitalisation rates in coal mining counties
N17, N18, N19 (Acute and unspecific kidney failure, chronic kidney disease)	n [56]	Population in 73 USA counties	OR = 0.997(0.994-0.999) hospitalisation rates in coal mining counties
N25, N26.9, N27 (Unspecific contracted and small kidney, renal sclerosis)	n [56]	Population in 73 USA counties	OR = 0.997(0.994-0.999) hospitalisation rates in coal mining counties
Chapter: Certain conditions originating in the perinatal period			
P07.0, P07.1 (Extremely and other low birth weight new-born)	y [55]	Mothers in 29 USA counties	OR = 1.16(1.08-1.25) *p* < 0.0002, OR = 1.14(1.04-1.25) *p* < 0.0033; cases in coal mining counties
Chapter: Congenital malformations, deformations and chromosomal abnormalities (Q00-Q99)	y [50, 54] **/** NS [50]	New-borns in 86 USA counties	Adjusted PRR ranging 1.10-1.63 new-born hospitalisations in coal mining counties (adjusted for socioeconomic variables). Crude PRR ranging 1.43-2.39 *p* < 0.001 new-born hospitalisations in coal mining counties. NS: Adjusted PRR ranging 1.01-1.08 *p* > 0.05 new-born hospitalisations in coal mining counties (adjusted by type and group of hospital)
Q00-07 (Congenital malformations of the nervous system)	y [54]	New-borns in 86 USA counties	Adjusted PRR = 1.36(1.11-1.67) new-born hospitalisations in coal mining counties
Q20-34 (Congenital malformations of the circulatory and respiratory systems)	y [54]	New-borns in 86 USA counties	Adjusted PRR = 1.93(1.73-2.15) new-born hospitalisations in coal mining counties
Q35-45 (Congenital malformations of the digestive system)	y [54]	New-borns in 86 USA counties	Adjusted PRR = 1.41(1.17-1.71) new-born hospitalisations in coal mining counties
Q50-64 (Congenital malformations of genitals and urinary system)	y [54]	New-borns in 86 USA counties	Adjusted PRR = 1.35(1.19-1.54) new-born hospitalisations in coal mining counties
Q65-79 (Congenital malformations and deformations of the musculoskeletal system)	y [54]	New-borns in 86 USA counties	Adjusted PRR = 1.30(1.20-1.41) new-born hospitalisations in coal mining counties
Q80-89 (Other congenital malformations)	y [54]	New-borns in 86 USA counties	Adjusted PRR = 1.13(1.04-1.23) new-born hospitalisations in coal mining counties

Increased risk y/n: one or more risk measure increased/non-increased in exposed versus non-exposed populations. *r* regression coefficient, *OR* odds ratio, *RR* relative risk, *PRR* prevalence rate ratio

^a^ NS: Not significant

^b^ Disparities found in different exposed sub-groups

All risk measures provided in additional material [see Additional file 4]

### Mortality

Significant risk measures of mortality were found in 12 studies of mortality [31–44] and one study of mortality and morbidity [30]. Increased risk of mortality in exposed populations was found for 7 grouped ICDs (blocks, chapters and groups of combined chapters) and 64 ICD categories in the following chapters: neoplasms, diseases of the circulatory system, diseases of the respiratory system, diseases of the genitourinary system, and external causes of morbidity. Non-increased risk of mortality in exposed populations was found for 2 grouped ICDs and 28 ICD categories in the chapters: neoplasms, diseases of the circulatory system, diseases of the respiratory system, diseases of the genitourinary system, and external causes of morbidity (Table 3).

One of the studies [34] found non-significant increased risk of mortality in exposed populations by all causes of disease although the article was subjected to erratum after the results were disputed [45]. Two of the studies [32, 42] found both increased and non-increased risk of mortality in exposed populations for ICD categories in 4 chapters of the ICD (diseases of the circulatory system, diseases of the respiratory system, diseases of the genitourinary system, external causes of disease), and one group of ICD chapters (all combined internal and external causes). These disparities were found in some but not all exposed population sub-groups included in the studies. One of the studies [31] did not calculate risk and rather identified increased mortality for ICDs in three chapters (neoplasms, diseases of the respiratory system, and external causes of morbidity) from a graphical analysis of rates in exposed /non-exposed populations.

Exposed populations were residents of USA counties with occurrence of coal mining (coal mining counties) in all mortality studies with exception of one study [33] that included populations in Spanish towns in proximity to coal mining.

### Mortality and morbidity

Three studies of live and still births found increased and non-increased risk measures with different significance for association of residence and proximity to coal mining with congenital malformations and chromosomal abnormalities. Liao et al. [46] found increased risk in mothers resident in coal mining counties, and identified increased rates in populations in proximity to coal mining (measure not provided) [47]. Gu et al. [48] found higher (although non-significant difference) mortality rates in villages closer to coal mining plants compared to control populations (Table 3).

### Morbidity

Significant risk measures of morbidity were found in 10 studies of morbidity [49–58] and one study of mortality and morbidity [30]. Increased risk of morbidity in exposed populations was found for 9 grouped ICDs (blocks and chapters) and 21 ICD categories in the following chapters: neoplasms, endocrine, nutritional and metabolic diseases, diseases of the eye and adnexa, diseases of the circulatory systems, diseases of the respiratory system, diseases of the skin and subcutaneous tissue, conditions of the perinatal period, and congenital malformations and chromosomal abnormalities. Non-increased risk of morbidity in exposed populations was found for 18 ICD categories in the chapters: diseases of the eye and adnexa, diseases of the respiratory and genitourinary systems, and diseases of the skin and subcutaneous tissue (Table 4).

One of the studies [49] found non-significant risk (increased) of morbidity by diseases of the circulatory system (whole chapter) in exposed populations. One study [50] found non-significant risk (increased) of congenital malformations in new-borns to mothers resident in coal mining counties after adjustment by groups and type of hospitals. Two of the studies [57, 58] found that children in proximity to coal mining have both increased and non-increased risk of ICD categories in three chapters of the ICD (diseases of the eye and adnexa; diseases of the skin and subcutaneous tissue, diseases of the circulatory system). These disparities were found in one out of five exposed sub-groups included in the two studies.

Exposed populations were residents of USA coal mining counties and USA minor civil divisions in all morbidity studies, with exception of two studies [57, 58] of communities in proximity to coal mining in northern England.

### Covariates

All of the studies with the exception of two [31, 50] included covariates for adjustment in the statistical analysis. Sociodemographic variables included socioeconomic indicators, race/ethnicity, education level, and health-care access. Environmental variables included soil or land cover type, proximity to rivers and faults, elevation, exposure to pesticides, levels of heavy metals in soil, and indoor and outdoor pollution. Twenty five (89%) of the studies adjusted for sociodemographic variables, 13 (46%) for smoking, 6 (21%) for obesity and/or overweight, 5 (19%) for other comorbidities or family history of comorbidity, and 4 (14%) for environmental variables.

### Studies design and methods

All studies followed an ecological design (i.e. one or more variables included, at the group level). Regression analyses were used in 23 (82%) of the studies. Six of the studies (21%) included both regression and spatial analyses. Four (14%) of the studies conducted only spatial analyses (Table 2).

The ecological units were assigned according to administrative divisions (e.g., counties or towns) and geographical points. All studies from the USA used county as the ecological unit with the exception of one study [52] that used minor civil divisions (i.e. administrative divisions of a county). Two studies in China [46, 47] used points in a grid designed for the study, the study in Spain [33] used towns, and one study in China [48] used buffer distance units.

### Critical appraisal of the studies

The studies were assessed using a modified scale of a checklist designed for quality assessment of ecological studies [29]. None of the studies scored low relevant: seven (25%) of the studies scored high and 21 (75%) scored medium in the assessment scale. The scores for each item are presented in Table 5.

**Table 5:** Critical appraisal of selected studies. Modified scale from Dufault and Klar [29]

Author (year)	Study design and focus			Statistical methodology				Quality of reporting			Score
	Sample size	Level of inference	Pre-specification of ecological units	Validity of statistical inferences	Use of covariates	Spatial effects	Proper adjustment for covariates	Statement of study design	Justification of study design	Discussion of cross-level bias and limitations	Points
Liao et al. (2016)	2	1	1	0	0	1	0	1	1	0	7
Woolley et al. (2015)	2	1	0	0	0	0	0	1	0	1	5
Talbott et al. (2015)	2	1	0	2	1	1	0	1	0	1	9
Mueller et al. (2015)	2	1	0	2	1	1	0	1	1	1	10
Lamm et al. (2015)	2	1	0	2	0	0	0	0	0	0	5
Buchanich et al. (2014)	1	1	0	2	1	0	1	0	0	1	7
Brink et al. (2014)	2	1	0	2	1	0	0	1	0	0	7
Liu et al. (2013)	2	1	0	2	1	0	1	0	1	0	8
Fernandez-Navarro et al. (2012)	2	1	0	2	1	1	0	1	0	1	9
Ahern and Hendryx (2012)	2	1	0	2	1	0	0	1	1	0	8
Borak et al. (2012)	2	1	0	2	1	0	0	0	0	0	6
Ahern et al. (2011)	2	1	0	2	1	1	1	1	1	1	11
Esch and Hendryx (2011)	2	1	0	2	1	0	0	1	1	1	9
Christian et al. (2011)	2	1	0	2	1	1	0	0	1	0	8
Hendryx (2011)	2	1	0	2	1	0	0	0	0	1	7
Ahern, MacKay and Hamilton (2011)	2	1	0	2	1	0	1	0	0	0	7
Liao et al. (2010)	2	1	1	2	1	1	0	1	1	0	10
Hendryx et al. (2010a)	2	1	0	2	1	1	0	1	0	1	9
Hendryx, Fedorko and Halverson (2010)	2	1	0	2	1	0	0	1	0	0	7
Hitt and Hendryx (2010)	2	1	0	0	0	1	0	0	0	1	5
Hendryx (2009)	2	1	0	2	1	0	0	0	0	1	7
Hendryx and Ahern (2009)	2	1	0	2	1	0	0	0	0	0	6
Hendryx (2008)	2	1	0	2	1	0	0	1	0	0	7
Hendryx et al. (2008)	2	1	0	2	1	0	0	0	0	0	6
Hendryx et al. (2007)	2	1	0	2	1	0	1	1	0	1	9
Gu et al. (2007)	2	1	0	0	1	1	0	0	1	0	6
Howel et al. (2001)	2	1	0	2	1	0	1	0	0	0	7
Pless-Mulloli et al. (2000)	2	1	0	2	1	0	1	0	0	0	7

## Discussion

The ICD diagnosis codes presented in this systematic review unify health outcomes found in diverse studies in a single standard classification. We mapped ICDs to reported health outcomes in studies of population in proximity or resident of coal mining areas. These populations have increased risk of a wide spectrum of diseases encompassing 78 ICD categories and 9 groups of ICDs, classified in 10 out of 21 chapters of the ICD-10-CM. The ICDs were reported by studies of mortality and/or morbidity designed to assess ecological exposures, namely ecological studies, in the USA, UK, Spain and China.

### Scope of health outcomes and ICDs identified in the selected studies

The majority of the studies found increased risk of one or more ICD diagnosis categories in exposed versus non-exposed, especially for ICDs in the chapters neoplasms, diseases of the circulatory and respiratory systems, and congenital anomalies; that were reported in both mortality and morbidity studies. Two thirds of the studies found increased mortality by cancer in exposed populations and nearly 40% of all ICDs identified were ICD categories of cancer. Most of the mortality studies found higher risk of cancer of lung and colon, and all combined cancer, and two of the morbidity studies found increased risk of cancer of lung and colon. These results show a consistent association of coal mining with higher mortality and morbidity by cancer in populations near coal mining.

The association of coal mining with cancer has been documented in epidemiological studies of workers since the 1930s [5], and increased risk of cancer of lung and stomach has been evidenced in epidemiological studies of occupational exposed populations [5, 59, 60]. It should be noted that populations in studies of this review could include coalminers that were part of the chosen exposed communities. Given that most coalminers are men, almost all of the studies conducted statistical analyses adjusting by gender to segregate populations with and without occupational exposure. The results evidence an association of coal mining with cancer of lung and colon, adjusted by gender, in the exposed populations. Furthermore, the study of Fernandez-Navarro et al. found increased risk of colorectal and thyroid cancer in only women of populations in proximity to coal mining [33]. They also found that only men in these populations had increased risk of cancer of liver, brain and thyroid, a group of diagnoses for which there is scarce research from studies of workers. These findings suggest some association of coal mining with cancer of organs scarcely investigated in studies of both occupational and non-occupational exposed populations.

The other ICDs of cancer in this review include categories such as cancer of stomach, oesophagus, kidney, bladder, brain, leukaemia and cervical cancer. There is little research about these diseases in the general population, regarding their proximity to coal mining. Studies of coal miners have found increased risk of cancer of stomach, kidney and bladder [17]. Other studies in coal miners found increased risk of leukaemia [61] and cancer of brain [62] although the results were inconclusive and exposures could be related to electrical and magnetic fields rather than contact with sub products of the mining activities. For some ICD categories of cancer found in this review such as cancer of oesophagus, brain and meninges, thyroid, leukaemia and cervical cancer, we did not find evidence of their association with occupational exposures. The increased risk of these diverse types of cancer in the general population studied can indicate that cancer development follows different exposure pathways in communities resident, or in proximity of coal mining. Whereas in ecological studies other risk factors such as socioeconomic variables can account for effects at the group level of the analysis, all studies in this review conducted analyses adjusting for sociodemographic and other variables (with the exception of one study that adjusted only for type of hospitals [50]). The multiple ICDs of cancer identified in studies of this review reflect that cancer more than a disease in isolation is in fact many diseases -mostly chronic- of different organ systems. Since coal mining implies industrial activities prolonged for many years, eventually different exposure pathways can relate coal mining with biological insult on diverse organ systems that result in disease.

Exposure pathways need to be considered for the increased risk of congenital anomalies found in some of the studies. The five studies that included live or still births to mothers resident or in proximity of coal mining found increased risk of congenital anomalies, yet there was variation in the significance of the results. Two studies [46, 47] found increased risk of neural tube defects (NTD), i.e. congenital anomalies of the nervous system, and a third study found increased but non-significant, mortality by NTD in live and still births in populations close to coal mining [48]. These findings were consistent with a fourth study by Ahern et al. that found increased risk of hospitalisation by NTD and congenital anomalies in other four organ systems, as well as all combined congenital anomalies [54]. The fifth study (Lamm et al.) used the same sample of Ahern et al. (USA counties) and found non-significant (increased) risk of hospitalisation by all combined congenital anomalies, after adjustment by type and groups of hospitals [50]. The study of Lamm et al. however, did not adjust by socioeconomic variables. The combined evidence of these studies suggests that pregnant women resident or in proximity of coal mining have a higher risk of carrying pregnancies affected by congenital malformations or chromosomal abnormalities.

The possible exposure pathways related to congenital anomalies in populations in proximity to coal mining are not established although it is accepted that most congenital anomalies result from interaction between both genetic and environmental factors [63]. There is increasing evidence of the association of congenital anomalies with exposure to environmental risk factors. Increased risk of congenital anomalies was found in mothers with higher blood levels of environmental pollutants such as arsenic and cadmium in coal mining areas of China [64]. These results follow another study that reported higher blood concentrations of arsenic and cadmium from coal mining in pregnant women from the same region [65]. Increased rates of congenital malformations and adverse pregnancy outcomes have been associated with air pollution [66, 67] and there are established links between congenital anomalies and environmental tobacco smoke [68, 69]. Moreover, environmental exposures have been associated with low birth weight, a perinatal condition with physio-pathological mechanisms related to congenital anomalies [70, 71]. One of the morbidity studies in this review (Ahern et al.) found increased risk of low and extremely low birth weight in new-borns to mothers resident in coal mining counties [55]. The women included in the Ahern et al. study [55] were part of the same populations in the studies that found increased risk of congenital anomalies in the USA. This supports the plausibility of exposure pathways between coal mining and pathogenic effects on the foetus development and low birth weight.

Almost 40% of the mortality studies searched the association of coal mining with all combined causes of disease. Five of these studies found increased risk of mortality by all causes in people resident of USA open-cut coal mining counties and one more study identified increased rates of mortality in a comparative analysis [31]. Only one of the mortality studies found non-significant (increased) mortality by all combined causes in a re-analysis of data used in three other mortality studies in this review [34]. However the original authors disputed the methods and results, and the article was subjected to erratum [45]. All of these studies adjusted their analyses for socioeconomic variables and other covariates such as smoking and other co-morbidities. These findings consistently show that communities resident in USA coal mining counties bear a higher risk of general mortality compared to non-coal mining counties.

A smaller number of the studies found significant risk of diseases of the circulatory and respiratory systems in residents of coal mining areas. Esch and Hendryx found higher mortality by five ICDs of chronic cardiovascular diseases in exposed populations [36]. These findings were consistent with a study of Hendryx who found increased risk for the same ICD categories and other 16 ICD categories of diseases of the respiratory system [44]. This study however, also found non-increased risk of all of the ICD categories in different exposed subgroups of population. Whereas three studies of morbidity found increased risk of hospitalisations and medical consultations by respiratory diseases [56–58] the studies of Pless-Mulloli et al. [58] and Howel et al. [57] found non-increased rates of consultations in one out of five subgroups of exposed communities. From the results of these studies in the general population there is a significant association between coal mining and diseases of the circulatory and respiratory systems although there is not consistency in the studies’ findings to characterise the trend by these diseases in affected communities.

One of the studies [30] found non-increased risk of prostate cancer in exposed populations. Whereas no other study of this review included prostate cancer, these findings concur with results of studies in workers reviewed by Jenkins et al. [17]. We did not find published evidence to support biological plausibility of lower risk of prostate cancer in coal mining exposed populations. Nevertheless, a hypothetical protective effect of coal mining on prostate cancer has been explored previously in coalminers although the authors did not infer specific exposure pathways or hazards related to the lower risk [72]. On the other hand, Hendryx et al. found lower ORs of hospitalizations rates in residents of USA coal mining counties by 15 ICD categories of diseases of the genitourinary system [56]. In another study Hendryx found only increased mortality rates for some of these ICDs in similar populations [42], however these two studies differ in the sources of data (death certificates versus hospital records). These findings suggest a lower risk of diagnoses involving organs of the genitourinary system in residents of USA coal mining counties related with underlying mechanisms not yet investigated. Girschik et al. [72] conducted a systematic review of prostate cancer in studies of coal miners, measuring a combined effect size of 0.74 (95% CI 0.67 to 0.81), and suggested some occupational conditions as possible explanations. Nevertheless the proposed mechanisms do not extrapolate to the results in studies of this review, and their link with possible exposure pathways need to be investigated in further research.

### Methodological design of the studies and control of bias

The selection criteria in this systematic review did not include a specific study design yet all of the studies selected followed an ecological design (one or more variables grouped as rates or percentages). This circumstance is telling of some of the complexities faced by authors when addressing research of health outcomes associated with environmental exposures (e.g. exposures related with coal mining), that are eminently ecological. In many cases, the choice of this kind of design is the only way to conduct the research given the limitations to access protected data. A third of the authors of studies in this review noted that they follow an ecological design given restrictions to access individual data. The use of an ecological design in all selected studies, besides being an indicator of consistency seems to be adequate to approach important characteristics of the exposure, for example; the large regionalisation of coal mining areas, the indiscriminate impacts on surrounding populations and the validity of spatial and spatiotemporal analyses. Notwithstanding ecological studies have several limitations, especially the risk of ecological bias (i.e. confounding effect introduced by grouping variables). The PRISMA statement requires an assessment of risk of bias for studies included in systematic reviews, preferably with the use of standardised scales [27]. We used a modified assessment scale that has been applied in other reviews of ecological studies [73], instead of adapting standard scales more generally used (e.g. Newcastle-Ottawa scale). We consider this as the best approach to assess ecological studies, which include methodological characteristics such as samples based on ecological units and spatial analyses along with other statistical tests that cannot be evaluated with other assessment scales.

In the critical appraisal, all of the studies scored medium or highly relevant though there was important variability between scores of the scale’s criteria. The most pertinent issue was related to the use and adjustment for covariates in regression analyses to control risk of ecological bias [74]. Only seven of the studies conducted a proper adjustment for covariates as suggested for ecological studies [75]. All other studies did not include covariates in the regression analyses or included covariates measured as percentages instead of adjusting by age (as done for dependent variables). Given that calculation of age-adjusted rates for covariates is not always possible because of lack, or restrictions of data sources, this is a common limitation of ecological studies. However there were consistent results between studies that did proper adjustment for covariates and 17 studies that did not, while including similar exposed/non-exposed populations (e.g. the study of Buchanic et al. [32] and the studies of Henryx et al. [39], Hit and Hendryx [38], and Woolley et al. [31]). Likewise these results concurred with two of the studies conducted in regions other than the Appalachia [30, 33] indicating minor distortion (if any) of the results. Another aspect of the critical appraisal was related to the “quality of reporting” criterion. Only half of the studies did include an explicit statement about the study design, and discussed the risk of ecological bias. This presupposes a greater responsibility for the reader to be aware of the studies’ design and draw conclusions from their results. We analysed results of each study regarding the populations sampled and considering consistency between results of different studies. Since ecological studies are most useful to provide exploratory analysis and generate hypothesis, rather than establishing causal links [76] the quality assessment of the studies did not affect majorly our synthesis and interpretation of results. None of the studies scored low relevant and there were consistent results in two or more studies, for risk characterisation of ICD categories in most of the ICD chapters, as discussed in the previous section.

The results of studies selected in this review can contribute to hypothesis testing in further research. A good example is in the results of the study of Liu et al. who found significant increased levels of blood sugar in diabetic residents of USA coal mining counties after adjustment for covariates [52]. Peer review literature about diseases of the endocrine system in populations in proximity to coal mining is scarce and these findings suggest an association needed to be studied in other coal mining regions. In another study, Hendryx found increased risk of mortality for, amid other diseases, acute bronchitis, bronchiolitis and emphysema in residents of coal mining counties [42]. To the best of our knowledge, this is the first epidemiological study to measure a significant association of coal mining with these ICD categories in exposed populations. Whereas these findings can be corroborated with longitudinal studies, they are also complementary for research in the environmental health field: Inclusion of large groups of diagnosis categories can allow identification of underlying associations that cannot be measured in studies of more specific groups of diseases. Most of the studies in this review were designed to measure risk of diseases already studied for their association with coal mining, in part because of supporting evidence provided by studies in coalminers. The inclusion in the analysis of groups of disease beyond diagnosis categories restricted to results of previous research can evidence increased risk of unexpected health outcomes and lead to further research about different exposure pathways.

### Limitations

Our search criteria excluded studies for which ICD codes could not be assigned because the sources of health data were not validated on medical diagnoses. This restricted the inclusion of health conditions or disorders associated with coal mining detected in surveys and other non-medical assessments. We considered more relevant to focus on the validity of health outcomes and the use of a single classification standard. Studies of only coalminers were excluded. Since workers could be part of the population in proximity to coal mining, their exclusion could impact the ICDs to be identified. However studies of workers are usually designed to assess occupational exposures (e.g. vibration, use/not use of masks), and coalminers are mostly men, two characteristics that are not representative of the general population. We balanced the exclusion of studies of occupational exposed populations against the chance of selection bias of ICDs in populations in the vicinity of coal mining. All selected studies in the review followed an ecological design. The issues related to ecological studies treated in the discussion imply that the interpretation of results for individual studies must be contextualised with the populations sampled and the statistical analyses of each study. Given that the selected articles were published in English, we did not assess studies from other coal mining regions reported in other languages. Whereas there seems to be a significant regional concentration of studies, we could not identify ICDs associated with coal mining in regions other than the USA, Europe and China.

## Conclusions

There is consistent evidence of the association of coal mining with a wide spectrum of diseases, especially cancer and congenital anomalies, in populations resident or in proximity of the mining activities. The studies that have investigated these associations were designed to measure exposures at the group level thus other research methods such as individual-level and longitudinal studies can be integrated to provide further evidence of the exposure pathways. Although coal mining is undertaken worldwide, the majority of the studies have been conducted in a few countries. More epidemiological studies of populations in coal mining areas are needed to expand the results of this review to most geographical regions.

## Additional files

Additional file 1: Checklist of items of the PRISMA protocol addressed. (DOCX 26 kb)

Additional file 2: Search strategies carried on Pubmed, Embase and Scopus. (DOCX 17 kb)

Additional file 3: Data extraction form, Items of data collected from the eligible studies. (DOCX 18 kb)

Additional file 4: All measures of risk and covariates reported in the eligible studies. (DOCX 167 kb)

## Abbreviations

ICD: International classification of diseases
ICD-10-CM: International classification of diseases 10th edition, clinical modification
