# Supplementary material for: Mortality and morbidity in populations in the vicinity of coal mining: a systematic review
Source: BMC Public Health. 2018 Jun 11;18:721. doi: 10.1186/s12889-018-5505-7 (PMC5996462; doi:10.1186/s12889-018-5505-7)
Supplement: Supplementary file 2 — Search strategies carried on Pubmed, Embase and Scopus. (DOCX 17 kb) [file 12889_2018_5505_MOESM2_ESM.docx]

Additional file 2.

Search strategy, Pubmed:

(((((((((((((((prevalence[Title/Abstract]) OR incidence[Title/Abstract]) OR mortality[Title/Abstract]) OR morbidity[Title/Abstract]) OR "health impact*"[Title/Abstract]) OR "health outcome*"[Title/Abstract]) OR "International Classification of Diseases"[Title/Abstract]) OR icd[Title/Abstract]) OR hospitalization*[Title/Abstract]) OR hospitalisation*[Title/Abstract]) OR "hospital discharge*"[Title/Abstract]) OR "hospital separation*"[Title/Abstract]) OR disease*[Title/Abstract]) OR death*[Title/Abstract])) AND (((coal[Title/Abstract]) OR "coal min*"[Title/Abstract]) OR "Coal Mining"[Mesh])

Search strategy, Embase:

‘coal min*’:ab,ti OR ‘coal mining’:exp AND prevalence:ab,ti OR incidence:ab,ti OR mortality:ab,ti OR morbidity:ab,ti OR 'health impact*':ab,ti OR 'health outcome*':ab,ti OR 'international classification of diseases':ab,ti OR icd:ab,ti OR hospitalization*:ab,ti OR hospitalisation*:ab,ti OR 'hospital discharge*':ab,ti OR 'hospital separation*':ab,ti OR disease*:ab,ti OR death*:ab,ti

Search strategy, Scopus:

( ( ABS ( "coal min*" ) OR TITLE ( "coal min*" ) ) ) AND ( ( ABS ( prevalence ) OR TITLE ( prevalence ) OR ABS ( incidence ) OR TITLE ( incidence ) OR ABS ( mortality ) OR TITLE ( mortality ) OR ABS ( morbidity ) OR TITLE ( morbidity ) OR ABS ( "health impact" ) OR TITLE ( "health impact" ) OR ABS ( "health outcome" ) OR TITLE ( "health outcome" ) OR ABS ( "international classification of diseases" ) OR TITLE ( "international classification of diseases" ) OR ABS ( icd ) OR TITLE ( icd ) OR ABS ( hospitalization ) OR TITLE ( hospitalization ) OR ABS ( hospitalisation ) OR TITLE ( hospitalisation ) OR ABS ( "hospital discharge" ) OR TITLE ( "hospital discharge" ) OR ABS ( "hospital separation" ) OR TITLE ( "hospital separation" ) OR ABS ( disease ) OR TITLE ( disease ) OR ABS ( death ) OR TITLE ( death ) ) )
