# Supplementary material for: Mortality and morbidity in populations in the vicinity of coal mining: a systematic review
Source: BMC Public Health. 2018 Jun 11;18:721. doi: 10.1186/s12889-018-5505-7 (PMC5996462; doi:10.1186/s12889-018-5505-7)
Supplement: Supplementary file 3 — Data extraction form, Items of data collected from the eligible studies. (DOCX 18 kb) [file 12889_2018_5505_MOESM3_ESM.docx]

Additional file 3. Data extraction form

| **Item** | **Description** |
| --- | --- |
| Title | As published |
| Authors | As published |
| Year | Year of publication |
| Funding source | Name of funding sources, description of activity such as education, industry, government |
| Country | Country of the exposed/non-exposed populations included in the study |
| Region | Geographical region of the exposed/non-exposed populations |
| Ecological units | Units of grouping exposed/non-exposed for statistical analyses |
| Type of Study | Mortality, morbidity, mortality and morbidity |
| Methods | List of the analytical methods used to produce the results of the study |
| Health outcomes scoped in the design | List of the health outcomes will be studied, as stated in the methods |
| Risk measures | Results of analysis and/or measures resulting from each of the methods in the previous point |
| ICD already in the report | The report shows results already classified as ICD9 or ICD10 (y/n) |
| ICD categories / code and description (as ICD10-CM) | List of ICD single categories found in the results / code, description, chapter in the ICD10-CM |
| ICD blocks / code and description (as ICD10-CM) | List of ICD blocks of categories found in the results / code, description, chapter in the ICD10-CM |
| ICD whole chapters / code and description (as ICD10-CM) | List of ICD whole chapters found in the results / code, description, chapter in the ICD10-CM |
| ICD combined (in more than one chapter) / code and description (as ICD10-CM) | List of ICD in more than one chapter, found in the results / code, description, chapter in the ICD10-CM |
| Covariates | List of covariates included in the analyses and results. Categories: smoking, sociodemographic, environmental, obesity/overweight, other co-morbidities |
