# Supplementary material for: Mortality and morbidity in populations in the vicinity of coal mining: a systematic review
Source: BMC Public Health. 2018 Jun 11;18:721. doi: 10.1186/s12889-018-5505-7 (PMC5996462; doi:10.1186/s12889-018-5505-7)
Supplement: Supplementary file 4 — All measures of risk and covariates reported in the eligible studies. (DOCX 167 kb) [file 12889_2018_5505_MOESM4_ESM.docx]

Additional file 4. All measures of risk reported in the selected studies

Conventions: RR=relative risk; OR=odds ratio; rR=rate ratio; pR= prevalence ratio; PRR=prevalence rate ratio; adj=adjusted; un-adj=unadjusted; Mr= mortality rate; AAMr= age adjusted Mr; Hr=hospitalization rate, Ir=Incidence rate, CM= coal mining; CMC= coal mining counties; MTM= mountain top mining; r (SE)= regression coefficient (Standard Error), PCC=Pearson correlation coefficient, GP=General practitioner. Non-increased risk measures in red

| **Class of study** | **ICD** | **Values** | **Covariates** |
| --- | --- | --- | --- |
| Mortality | C00-C97 (All malignant neoplasms) | **adj.RR Mr category CMC/Mr non-CMC** (Buchanich et al. 2014)  =1.05(1.03-1.08) p<0.01: underground&surface-CMC 82.6-209.6Mtons;  =1.06(1.03-1.09) p<0.01: underground&surface-CMC >209Mtons  =1.06(1.04-1.09) p<0.01: surface-CMC <4.2Mtons;  =1.03(1.01-1.06) p<0.01: surface-CMC 4.2-15.3Mtons;  =1.04(1.01-1.07) p<0.01: surface-CMC >68.1Mtons;  = 1.05(1.02-1.08) p<0.01: underground-CMC 1-4.2Mtons;  =1.04(1.01-1.07) p<0.01: underground-CMC 4.2-13.6Mtons;  =1.05(1.02-1.08) p<0.01: underground-CMC >13.6Mtons  =1.06(1.04-1.09) p<0.001: surface-CMC 20-years-lag  =1.01(1.01-1.01) p<0.001: underground&surface-CMC 10 years lag (2000-2009)  =1.03(1.02-1.04) p<0.001: surface-CMC 10-years-lag (2000-2009)  =1.06(1.03-1.09) p<0.01: surface-CMC 10-years-lag (1990-1999) | Gender, sociodemographic, environmental, obesity/overweight |
|  |  | **r (SE) increase AAMr in non-metropolitan-CMC by coal-production** (Hendryx, Fedorko, and Halverson 2010)  =7.14(2.96) p<0.02: low-production  =5.62(2.73) p<0.04: high-production | Gender, sociodemographic, smoking |
|  |  | **r (standardized/adjustedR2) increase AAMr in CMC** (Hendryx, Fedorko, and Anesetti-Rothermel 2010)  =0.369/0.36 p<0.003 | Gender, sociodemographic, Smoking |
|  |  | **PCC Mr in CMC with mining index** (Hitt and Hendryx 2010)  =0.42 p<0.01: 1000Tons/Km2  =0.51 p<0.01: CM-index | Sociodemographic, smoking |
|  | C15 (Cancer esophagus) | **r (SE) increase AAMr in CMC other-than-MTM** (Ahern and Hendryx 2012)  =0.766(0.353) p<0.05: (between 2003-2007) | Gender, sociodemographic, smoking, obesity/overweight |
|  | C16 (Cancer stomach) | **r (SE) increase AAMr in MTM-CMC** (Ahern and Hendryx 2012)  =0.935(0.482) p<0.05: (between 2003-2007) | Gender, sociodemographic, smoking, obesity/overweight |
|  | C18, C19, C20, C21 (Colorectal, anal cancer) | **r (SE) increase AAMr in MTM-CMC** (Ahern and Hendryx 2012)  =3.0(0.99) P<0.01: (between 2003-2007) | Gender, sociodemographic, smoking, obesity/overweight |
|  |  | **RR dead-in-towns<5Km-to-underground-CM/dead-in-towns>5Km-to-underground-CM** (Fernandez-Navarro et al. 2012)  = 1.27(1.12-1.44): men&women  = 1.31(1.13-1.52): men | Sociodemographic |
|  | C22 (Cancer liver) | **RR dead-in-towns<5Km-to-surface-CM/dead-in-towns>5Km-to-surface-CM** (Fernandez-Navarro et al. 2012)  = 1.69(1.09-2.63): men | Sociodemographic |
|  |  | **r (SE) increase AAMr** **in MTM-CMC** (Ahern and Hendryx 2012)  =0.788(0.395) p<0.05: (between 1999-2002) | Gender, sociodemographic, smoking, obesity/overweight |
|  | C32 (Cancer Larynx) | **r (standardized/adjustedR2) increase AAMr in CMC** (Hendryx, Fedorko, and Anesetti-Rothermel 2010)  =0.367/0.42 p<0.002 | Gender, sociodemographic, Smoking |
|  | C33 (Cancer Trachea) | **r (standardized/adjustedR2) increase AAMr in CMC** (Hendryx, Fedorko, and Anesetti-Rothermel 2010)  =0.367/0.42 p<0.002 | Gender, sociodemographic, Smoking |
|  | C34 (Cancer Bronchus and Lung) | **r (SE) increase AAMr in MTM-CMC** (Ahern and Hendryx 2012)  =10.3(2.6) p<0.01: (between 1999-2002)  =4.89(2.44) p<0.05: (between 2003-2007) | Gender, sociodemographic, smoking, obesity/overweight |
|  |  | **RR dead-in-towns<5Km-to-CM/dead-in-towns>5Km-to-CM (surface CM)** (Fernandez-Navarro et al. 2012)  = 1.22(1.01-1.49) men&women  = 1.29(1.05-1.59) men | Sociodemographic |
|  |  | **r (standardized/adjustedR2) increase of AAMr in CMC** (Hendryx, Fedorko, and Anesetti-Rothermel 2010)  =0.367/0.42 p<0.002 | Gender, sociodemographic, Smoking |
|  |  | **adj.r increase AAMr** **in Appalachian-CMC** (Hendryx, O'Donnell, and Horn 2008)  =5.60 p<0.008: surface-CM  =4.55 p<0.024: underground-CM  =3.72 p<0.036: surface&underground-CM | Gender, sociodemographic |
|  | C30-C39 (Cancer respiratory/intrathoracic organs) | **PCC Mr in CMC with mining index** (Hitt and Hendryx 2010)  =0.47 p<0.01: 1000Tons/Km2  =0.53 p<0.01: CM-index | Sociodemographic, smoking |
|  |  | **Mr in CMC** (Woolley et al. 2015)  =increased rates in males and females, in a graphical analysis and comparative t-test |  |
|  | C43 (Melanoma) | **r (standardized/adjustedR2) increase AAMr in CMC** (Hendryx, Fedorko, and Anesetti-Rothermel 2010)  =0.441/0.16 p<0.002  **r (standardized/adjustedR2) increase AAMr by tons-coal-produced**  =0.324/0.1 p<0.02 | Gender, sociodemographic, Smoking |
|  | C53 (cervical cancer) | **r (SE) increase AAMr in MTM-CMC** (Ahern and Hendryx 2012)  =0.699(.325) p<0.05: (between 1999-2002) | Gender, sociodemographic, smoking, obesity/overweight |
|  | C67 (Cancer Bladder) | **r (SE) increase AAMr in MTM-CMC** (Ahern and Hendryx 2012)  =1.33(.438) p<0.01: (between 2003-2007) | Gender, sociodemographic, smoking, obesity/overweight |
|  | C70 (Cancer meninges) | **r (standardized/adjustedR2) increase AAMr in CMC** (Hendryx, Fedorko, and Anesetti-Rothermel 2010)  =0.441/0.16 p<0.002  **r (standardized/adjustedR2) increase of AAMr** **by tons-coal-produced**  =0.324/0.1 p<0.02 | Gender, sociodemographic, Smoking |
|  | C71 (Brain cancer) | **r (standardized/adjustedR2) increase AAMr in CMC** (Hendryx, Fedorko, and Anesetti-Rothermel 2010)  =0.441/0.16 p<0.002  **r (standardized/adjustedR2) increase of AAMr** **by tons-coal-produced**  =0.324/0.1 p<0.02 | Gender, sociodemographic, Smoking |
|  |  | **RR of dead-in-towns<5Km-to-CM/dead-in-towns>5Km-to-CM (surface CM)** (Fernandez-Navarro et al. 2012)  = 1.75(1.19-2.57) men | Sociodemographic |
|  | C72 (Cancer spinal cord, cranial nerves, other central nervous system) | **r (standardized/adjustedR2) increase of AAMr in CMC** (Hendryx, Fedorko, and Anesetti-Rothermel 2010)  =0.441/0.16 p<0.002  **r (standardized/adjustedR2) increase of AAMr by tons-coal-produced**  =0.324/0.1 p<0.02 | Gender, sociodemographic, Smoking |
|  | C73 (Thyroid cancer) | **RR of dead-in-towns<5Km-to-CM/dead-in-towns>5Km-to-CM (underground CM)** (Fernandez-Navarro et al. 2012)  = 1.77(1.15-2.71) men&women  = 2.05(1.01-4.13) men  = 1.70(1.02-2.84) women | Sociodemographic |
|  | C81 (Hodgkin lymphoma) | **r (standardized/adjustedR2) increase of AAMr in CMC** (Hendryx, Fedorko, and Anesetti-Rothermel 2010)  =0.441/0.16 p<0.002  **r (standardized/adjustedR2) increase of AAMr by tons-coal-produced**  =0.324/=0.1 p<0.02 | Gender, sociodemographic, Smoking |
|  | C82, C83, C84, C85 (Follicular/non-follicular, mature T/NK-cell, other specified/unspecified non-Hodgkin lymphomas) | **r (standardized/adjustedR2) increase of AAMr in CMC** (Hendryx, Fedorko, and Anesetti-Rothermel 2010)  =0.441/0.16 p<0.002  **r (standardized/adjustedR2) increase of AAMr by tons-coal-produced**  =0.324/=0.1 p<0.02 | Gender, sociodemographic, Smoking |
|  | C88 (Malignant immunoproliferative-diseases, other B-cell lymphomas) | **r (standardized/adjustedR2) increase of AAMr in CMC** (Hendryx, Fedorko, and Anesetti-Rothermel 2010)  =0.441/0.16 p<0.002  **r (standardized/adjustedR2) increase of AAMr by tons-coal-produced**  =0.324/=0.1 p<0.02 | Gender, sociodemographic, Smoking |
|  | C90 (Multiple myeloma, plasmacytoma) | **r (standardized/adjustedR2) increase of AAMr in CMC** (Hendryx, Fedorko, and Anesetti-Rothermel 2010)  =0.441/0.16 p<0.002  **r (standardized/adjustedR2) increase of AAMr by tons-coal-produced**  =0.324/=0.1 p<0.02 | Gender, sociodemographic, Smoking |
|  | C91, C92, C93, C94, C95 (Leukemias) | **r (SE) increase of AAMr in MTM-CMC** (Ahern and Hendryx 2012)  =1.102(.554) p<0.05: (between 2003-2007) | Gender, sociodemographic, smoking, obesity/overweight |
|  |  | **r (standardized/adjustedR2) increase of AAMr in CMC** (Hendryx, Fedorko, and Anesetti-Rothermel 2010)  =0.441/0.16 p<0.002  **r (standardized/adjustedR2) increase of AAMr by tons-coal-produced**  =0.324/=0.1 p<0.02 | Gender, sociodemographic, Smoking |
|  | C96 (Unspecified cancer of lymphoid, hematopoietic tissue) | **r (standardized/adjustedR2) increase of AAMr in CMC** (Hendryx, Fedorko, and Anesetti-Rothermel 2010)  =0.441/0.16 p<0.002  **r (standardized/adjustedR2) increase of AAMr by tons-coal-produced**  =0.324/=0.1 p<0.02 | Gender, sociodemographic, Smoking |
|  | I00-I78 (All diseases circulatory system, except unclassified/unspecific) | **r (SE)** **increase of AAMr in metropolitan-high-CMC** (Hendryx, Fedorko, and Halverson 2010)  =14.32(6.61) p<0.03  =5.17 (5.97) p<0.39 | Gender, sociodemographic, Smoking |
|  | I10 (Essential HTA) | **adj.RR AAMr CMC/AAMr non-CMC** (Hendryx 2009)  =1.28(1.25-1.30): Males(Appalachian-CMC>4Mtons)  =1.18(1.15-1.21): Females(Appalachian-CMC>4Mtons)  =1.06(1.04-1.08): Males(Appalachian-CMC<4Mtons)  *=0.96(0.94-0.98): Males(non-Appalachian-CMC)*  *=0.97(0.95-0.99): Females(non-Appalachian-CMC)* | Gender, sociodemographic, Smoking |
|  |  | **r increase AAMr in MTM-CMC** (Esch and Hendryx 2011)  =16.9(7.5) p<0.03  **r increase AAMr in CMC by log-of-tons-mined**  =11.4(5.5) p<0.05 | Gender, sociodemographic, obesity/overweight |
|  | I11 (Hypertensive Heart disease) | **adj.RR AAMr category CMC/AAMr non-CMC** (Hendryx 2009)  =1.28(1.25-1.30): Males(Appalachian-CMC>4Mtons)  =1.18(1.15-1.21): Females(Appalachian-CMC>4Mtons)  =1.06(1.04-1.08): Males(Appalachian-CMC<4Mtons)  *=0.96(0.94-0.98): Males(non-Appalachian-CMC)*  *=0.97(0.95-0.99): Females(non-Appalachian-CMC)* | Gender, sociodemographic, Smoking |
|  |  | **r increase AAMr in MTM-CMC** (Esch and Hendryx 2011)  =16.9(7.5) p<0.03  **r increase AAMr in CMC by log-of-tons-mined**  =11.4(5.5) p<0.05 | Gender, sociodemographic, obesity/overweight |
|  | I12 (Hypertensive chronic kidney disease) | **adj.RR AAMr category CMC/AAMr non-CMC** (Hendryx 2009)  =1.28(1.25-1.30): Males(Appalachian-CMC>4Mtons)  =1.18(1.15-1.21): Females(Appalachian-CMC>4Mtons)  =1.06(1.04-1.08): Males(Appalachian-CMC<4Mtons)  *=0.96(0.94-0.98): Males(non-Appalachian-CMC)*  *=0.97(0.95-0.99): Females(non-Appalachian-CMC)* | Gender, sociodemographic, Smoking |
|  | I13 (Hypertensive heart&chronic kidney disease) | **r of increase AAMr** **in MTM-CMC** (Esch and Hendryx 2011)  =16.9(7.5) p<0.03  **r increase AAMr in CMC by log-of-tons-mined**  =11.4(5.5) p<0.05 | Gender, sociodemographic, obesity/overweight |
|  | I21 (ST elevation&non-ST myocardial infarction) | **adj.RR AAMr category CMC/AAMr non-CMC** (Hendryx 2009)  *=0.89(0.87-0.91): Males(Appalachian-CMC>4Mtons)*  *=0.95(0.93-0.97): Females(Appalachian-CMC>4Mtons)*  *=0.95(0.93-0.97): Males(Appalachian-CMC<4Mtons)*  *=0.97(0.94-0.99): Females(Appalachian-CMC<4Mtons)*  =1.06(1.04-1.08): *Males(non-Appalachian-CMC)*  =1.10(1.08-1.12): *Females(non-Appalachian-CMC)* | Gender, sociodemographic, Smoking |
|  |  | **r of increase AAMr** **in MTM-CMC** (Esch and Hendryx 2011)  =16.9(7.5) p<0.03  **r increase AAMr in CMC by log-of-tons-mined**  =11.4(5.5) p<0.05 | Gender, sociodemographic, obesity/overweight |
|  | I24 (Other acute ischemic heart diseases) | **adj.RR AAMr category CMC/AAMr non-CMC** (Hendryx 2009)  *=0.89(0.87-0.91): Males(Appalachian-CMC>4Mtons)*  *=0.95(0.93-0.97): Females(Appalachian-CMC>4Mtons)*  *=0.95(0.93-0.97): Males(Appalachian-CMC<4Mtons)*  *=0.97(0.94-0.99): Females(Appalachian-CMC<4Mtons)*  =1.06(1.04-1.08): Males(non-Appalachian-CMC)  =1.10(1.08-1.12): Females(non-Appalachian-CMC) | Gender, sociodemographic, Smoking |
|  | I25 (Chronic ischemic heart disease) | **adj.RR AAMr category CMC/AAMr non-CMC** (Hendryx 2009)  =1.28(1.25-1.30): Males(Appalachian-CMC>4Mtons)  =1.18(1.15-1.21): Females(Appalachian-CMC>4Mtons)  =1.06(1.04-1.08): Males(Appalachian-CMC<4Mtons)  *=0.96(0.94-0.98): Males(non-Appalachian-CMC)*  *= 0.97(0.95-0.99): Females(non-Appalachian-CMC)* | Gender, sociodemographic, Smoking |
|  |  | **r of increase AAMr in MTM-CMC** (Esch and Hendryx 2011)  =16.9(7.5) p<0.03  **r increase AAMr in CMC by log-of-tons-mined**  =11.4(5.5) p<0.05 | Gender, sociodemographic, obesity/overweight |
|  | I31, I33, (Endocarditis, other Pericardium-diseases) | **adj.RR AAMr category CMC/AAMr non-CMC** (Hendryx 2009)  *=0.89(0.87-0.91): Males(Appalachian-CMC>4Mtons)*  *=0.95(0.93-0.97): Females(Appalachian-CMC>4Mtons)*  *=0.95(0.93-0.97): Males(Appalachian-CMC<4Mtons)*  *=0.97(0.94-0.99): Females(Appalachian-CMC<4Mtons)*  =1.06(1.04-1.08): Males(non-Appalachian-CMC)  =1.10(1.08-1.12): Females(non-Appalachian-CMC) | Gender, sociodemographic, Smoking |
|  | I40 (Acute myocarditis) |  |  |
|  | I50 (Heart failure) | **r of increase AAMr in MTM-CMC** (Esch and Hendryx 2011)  =16.9(7.5) p<0.03  **r increase AAMr in CMC by log-of-tons-mined**  =11.4(5.5) p<0.05 | Gender, sociodemographic, obesity/overweight |
|  |  | **adj.RR AAMr category CMC/AAMr non-CMC** (Hendryx 2009)  *=0.89(0.87-0.91): Males(Appalachian-CMC>4Mtons)*  *=0.95(0.93-0.97): Females(Appalachian-CMC>4Mtons)*  *=0.95(0.93-0.97): Males(Appalachian-CMC<4Mtons)*  *=0.97(0.94-0.99): Females(Appalachian-CMC<4Mtons)*  =1.06(1.04-1.08): Males(non-Appalachian-CMC)  =1.10(1.08-1.12): Females(non-Appalachian-CMC) | Gender, sociodemographic, Smoking |
|  | I70 (Atherosclerosis) | **r of increase AAMr in MTM-CMC** (Esch and Hendryx 2011)  =16.9(7.5) p<0.03  **r increase AAMr in CMC by log-of-tons-mined**  =11.4(5.5) p<0.05 | Gender, sociodemographic, obesity/overweight |
|  | J00-J99 (All diseases respiratory system) | **r (SE)** **increase AAMr in high CMC** (Hendryx, Fedorko, and Halverson 2010)  = 6.29(1.79) p<0.001: metropolitan&non-metropolitan  = 9.81(2.32) p<0.0001: non-metropolitan | Gender, sociodemographic, Smoking |
|  |  | **Mr in CMC** (Woolley et al. 2015)  =increased rates in males and females, in a graphical analysis and comparative t-test |  |
|  | J12, J13, J14, J15, J16, J17, J18 (Pneumonias) | **adj.RR AAMr category CMC/AAMr non-CMC** (Hendryx 2009)  *=0.94(0.89-0.98): Males(Appalachian-CMC>4Mtons)*  *=0.89(0.84-0.94): Females(Appalachian-CMC>4Mtons)*  *=0.92(0.88-0.96): Males(Appalachian-CMC<4Mtons)*  *=0.92(0.87-0.97): Females(Appalachian-CMC<4Mtons)*  =1.05(1.01-1.09): Males(non-Appalachian-CMC)  =1.13(1.08-1.18): Females(non-Appalachian-CMC) | Gender, sociodemographic, Smoking |
|  | J20, J21 (Acute bronchitis, bronchiolitis) |  |  |
|  | J22 (Unspecified acute lower respiratory infection) |  |  |
|  | J40, J41, J42 (Bronchitis) | **adj.RR AAMr category CMC/AAMr non-CMC** (Hendryx 2009)  =1.07(1.04-1.10): Males(Appalachian-CMC>4Mtons)  =1.11(1.07-1.15): Females(Appalachian-CMC>4Mtons)  *=0.94(0.90-0.98): Females(Appalachian-CMC<4Mtons)*  =1.04(1.02-1.06): Males(non-Appalachian-CMC ) |  |
|  | J43, J44 (Emphysema, other chronic obstructive pulmonary disease) |  | Gender, sociodemographic, Smoking |
|  | J45 (Asthma) |  |  |
|  | N03, N04, N05 (Chronic nephritic, nephrotic syndromes) | **adj.RR AAMr category CMC/AAMr non-CMC** (Hendryx 2009)  =1.19(1.13-1.25): Males(Appalachian-CMC>4Mtons)  =1.13(1.06-1.21): Females(Appalachian-CMC>4Mtons)  =1.10(1.05-1.16): Males(Appalachian-CMC<4Mtons)  =1.14(1.07-1.21): Females(Appalachian-CMC<4Mtons)  =1.08(1.02-1.14): Females(non-Appalachian-CMC) | Gender, sociodemographic, Smoking |
|  | N17, N18, N19 (Kidney failure, chronic kidney-disease) |  |  |
|  | V00-V99 (All external causes) | **un-adj.RR Mr category CMC/Mr non-CMC** (Buchanich et al. 2014)  =1.10(1.02-1.19) p<0.01: underground&surface-CM>462Mtons  =1.12(1.04-1.21) p<0.01: surface-CM>68.1Mtons  *=0.91(0.85-0.99) p<0.01:* *underground-CM 38.6-73.6Mtons*  **adj.RR Mr category CMC/Mr non-CMC**  *=0.93(0.88-0.97) p<0.01: underground&surface-CM 38.6-102Mtons*  *=0.94(0.90-0.99) p<0.01:* *underground-CM 38.6-73.6Mtons* | Gender, sociodemographic, environmental, obesity/overweight |
|  |  | **Mr in CMC** (Woolley et al. 2015)  =increased rates in males and females, in a graphical analysis and comparative t-test |  |
|  | A00-Y89 (All internal and external causes) | **r of AAMr** **in CMC** (Hendryx and Ahern 2009)  r=31.06(7.46) p<0.0001 | Gender, sociodemographic, Smoking |
|  |  | **r (SE)** **increase AAMr by production** (Hendryx, Fedorko, and Halverson 2010)  =20.67(7.63) p<0.007: high-(metropolitan&non-metropolitan)  = 31.18(9.74) p<0.002: high-(non-metropolitan)  = 21.89(10.59) p<0.04: low-(non-metropolitan) | Gender, sociodemographic, Smoking |
|  |  | **adj.RR Mr category CMC/Mr non-CMC** (Buchanich et al. 2014)  *=0.97(0.95-0.99) p<0.01: underground&surface-CM 38.6-102Mtons*  =1.03(1.01-1.05) p<0.01: surface-CM<4.2Mtons;  *=0.97(0.95-0.99) p<0.01: underground-CM<38.6Mtons* | Gender, sociodemographic, environmental, obesity/overweight |
|  |  | **r (SE)** **increase AAMr CMC/ AAMr non-CMC** (Borak et al. 2012)  r=4.68(8.96) p<0.6003 SMR in coalmining counties | Gender, sociodemographic, Smoking |
|  |  | **Mr in CMC** (Woolley et al. 2015)  =increased rates in males and females, in a graphical analysis and comparative t-test |  |
|  | A00-R99 (All internal causes) | **adj.r increase AAMr in CMC>4Mtons** (Hendryx 2008)  =43.25 p<0.001: Appalachian-counties, surface-CM  =42.08 p<0.001: Appalachian-counties, underground-CM  =41.39 p<0.0004: Appalachian-counties, surface&underground-CM  =31.56 p<0.002: all-USA-CMC, underground-CM  =21.50 p<0.006: all-USA-CMC, surface&underground-CM | Gender, sociodemographic, Smoking |
|  |  | **adj.r (SE) increase AAMr in MTM-counties (Hendryx 2011)**  =63.0(13.8) p<0.002 | Gender, sociodemographic, Smoking, obesity/overweight |
| Morbidity | C34 (Cancer Bronchus and Lung) | **RR prevalence lung-cancer residents-of-CMC/prevalence lung-cancer residents-all-other-counties** (Christian et al. 2011)  =1.21 p<0.01: cluster1  =1.17 p<0.01: cluster2 | Gender, sociodemographic, smoking, |
|  | E10, E11, E13 (Diabetes-mellitus) | **r increase HbA1c (blood-sugar) previous-to-diagnosis, by coal-mining-area-density** (Liu et al. 2013)  =0.116(0.059) p<0.05  **r increase of HbA1c from-first-measure, by proximity-to-abandoned-coal-mine**  =0.124(0.056) p<0.01 | Gender, sociodemographic, obesity/overweight, other comorbidities |
|  | H57.9 (Unspecific disorder eyes) | **OR GP-consultations-exposed-communities/GP-consultations-control-communities** (Pless-Mulloli et al. 2000)  =1.40(1.15-1.70): communities 1,2,3,4  *=0.16(0.06-0.40): community 5* | Gender, sociodemographic, smoking, other comorbidities |
|  |  | **adj.OR GP-consultations-exposed-communities/GP-consultations-control-communities** (Howel, Pless-Mulloli, and Darnell 2001)  =1.43(1.20-1.70): communities 1,2,3,4  *=0.23(0.10-0.49): community 5* | Gender, sociodemographic, smoking, other comorbidities |
|  | I00-I99 (All diseases of the circulatory system) | **Adjusted Hr CMC/non-CMC by total coal production** (Talbott et al. 2015)  r=0.01 (0.01) p<0.28: men&women  r=0.01 (0.01) p<0.31: men  r=0.01 (0.01) p<0.26: women  **Adjusted Hr CMC/non-CMC by surface coal production**  r=0.04 (0.01) p<0.086: men&women  r=0.04 (0.01) p<0.088: men  r=0.05 (0.01) p<0.087: women | Sociodemographic,  smoking, obesity/overweight |
|  | I10, I11, I12, I13, I15 (Hypertensive diseases) | **OR of Hr/**$\sqrt[\boldsymbol{2}]{\boldsymbol{tons of coal produced*1000}}$ (Hendryx, Ahern, and Nurkiewicz 2007)  =1.003(1.001-1.005) | Gender, sociodemographic, other comorbidities |
|  | J00-J99 (All diseases respiratory system) | **r increase Hr by tons-of-surface-coal-produced** (Brink et al. 2014)  =0.064(0.025) p<0.014: men&women  =0.064(0.022) p<0.006: men  =0.063(0.029) p<0.032: women | Gender, sociodemographic, smoking |
|  | J40, J41, J42, J43, J44, J47 (Bronchitis, emphysema, chronic obstructive pulmonary disease, bronchiectasis) | **OR of Hr/**$\sqrt[\boldsymbol{2}]{\boldsymbol{tons of coal produced*1000}}$ (Hendryx, Ahern, and Nurkiewicz 2007)  =1.003(1.001-1.006) | Gender, sociodemographic, other comorbidities |
|  | J98.9 Unspecific respiratory disorders | **OR GP-consultations-exposed-communities/GP-consultations-control-communities** (Pless-Mulloli et al. 2000)  =1.42(1.13-1.79): communities 1,2,3,4  *=0.15(0.06-0.43): community 5* | Gender, sociodemographic, smoking, other comorbidities |
|  |  | **adjOR GP-consultations-exposed-group(s)/GP-consultations-control-group** (Howel, Pless-Mulloli, and Darnell 2001)  =1.47(1.22-1.78): groups 1,2,3,4  *=0.22(0.09-0.49): group 5* | Gender, sociodemographic, smoking, other comorbidities |
|  | L98.9 (Unspecific disorders skin) | **adjOR GP-consultations-exposed-group(s)/GP-consultations-control-group** (Howel, Pless-Mulloli, and Darnell 2001)  =1.43(1.20-1.70): groups 1,2,3,4  *=0.23(0.10-0.49): group 5* | Gender, sociodemographic, smoking, other comorbidities |
|  |  | **OR GP-consultations-exposed-communities/GP-consultations-control-communities** (Pless-Mulloli et al. 2000)  =1.40(1.15-1.70): communities 1,2,3,4  *=0.16(0.06-0.40): community 5* | Gender, sociodemographic, smoking, other comorbidities |
|  | N00.3, N00.8, N00.9, N01.3, N02.2, N03, N04, N05, N08 (Glomerular diseases) | **OR of Hr/**$\sqrt[\boldsymbol{2}]{\boldsymbol{tons of coal produced*1000}}$ (Hendryx, Ahern, and Nurkiewicz 2007)  *=0.997(0.994-0.999)* |  |
|  | N17, N18, N19 (Acute kidney failure, chronic kidney diseases) |  | Gender, sociodemographic, other comorbidities |
|  | N25, N26.9, N27 (Other disorders kidney and ureter) |  |  |
|  | P07.0, P07.1 (Low birth weight) | **OR to mothers-resident-in-CMC/mothers-resident-in-non-CMC** (Ahern, Mullett, et al. 2011)  =1.16(1.08-1.25) p<0.0002, high-CMC  =1.14(1.04-1.25) p<0.0033 moderate-CMC | Sociodemographic, smoking |
|  | Q00-Q99 (Congenital-malformations and chromosomal-abnormalities) | **adj.PRR MTM-CMC/pR non-CMC** (Ahern, Hendryx, et al. 2011)  =1.26(1.21-1.32)  **adj.PRR MTM-CMC/pR non-CMC**  =1.10(1.05-1.16)  **adj.PRR MTM-CMC/pR non-CMC**  =1.63(1.54-1.72)  **adj.PRR MTM-CMC/pR non-CMC**  =1.27(1.20-1.53) | Sociodemographic, smoking |
|  |  | **crude PRR MTM-CMC/pR non-MTM-CMC** (Lamm et al. 2015)  =1.43(1.36-1.52) p<0.001: all-hospitals  =1.43(1.35-1.51) p<0.001: group of 44 hospitals with greater than 1000 live births in MTM and Non-mining counties, combined  =2.39(2.15-2.65) p<0.001: group of 6 hospitals with greater than 1000 live births in MTM and Non-mining counties, separately  **adjusted** **PRR MTM-CMC/pR non-MTM-CMC**  *=1.08(0.97-1.20) p<0.16: group of 44-hospitals with greater than 1000 live births in MTM and Non-mining counties, combined*  *=1.01(0.89-1.14) p<0.87: group of 6-hospitals with greater than 1000 live births in MTM and Non-mining counties, separately* |  |
|  | Q00-07 (Congenital malformations nervous system) | **adj.PRR MTM-CMC/pR non-CMC** (Ahern, Hendryx, et al. 2011)  =1.36(1.11-1.67) | Sociodemographic, smoking |
|  | Q20-34 (Congenital malformations circulatory, respiratory systems) | **adj.PRR MTM-CMC/pR non-CMC** (Ahern, Hendryx, et al. 2011)  =1.93(1.73-2.15) | Sociodemographic, smoking |
|  | Q35-45 (congenital malformations digestive system) | **adj.PRR MTM-CMC/pR non-CMC** (Ahern, Hendryx, et al. 2011)  =1.41(1.17-1.71) | Sociodemographic, smoking |
|  | Q50-64 (congenital malformations genitals, urinary system) | **adj.PRR MTM-CMC/pR non-CMC** (Ahern, Hendryx, et al. 2011)  =1.35(1.19-1.54)  **adj.PRRR MTM-CMC/pR non-CMC**  =1.32(1.15-1.51) | Sociodemographic, smoking |
|  | Q65-79 (Congenital malformations musculoskeletal system) | **adj.PRR MTM-CMC/pR non-CMC** (Ahern, Hendryx, et al. 2011)  =1.30(1.20-1.41 | Sociodemographic, smoking |
|  | Q80-89 (Other congenital malformations) | **adj.PRR MTM-CMC/pR non-CMC** (Ahern, Hendryx, et al. 2011)  =1.13(1.04-1.23)  **adj.PRR MTM-CMC/pR non-CMC**  =1.12(1.03-1.22) | Sociodemographic, smoking |
| Mortality  and  morbidity | C00-C97 (All malignant neoplasms) | **r (standardized/adjustedR2) increase AAMr in CMC** (Mueller et al. 2015)  =0.39/0.46 p<0.001 | Sociodemographic, smoking |
|  | C18-C21 (Colorectal cancer) | **r increase Ir in CMT (recently-mined CM)** (Mueller et al. 2015)  =0.37 p<0.009  **r increase AAMr** **in CMT (recently-mined CM)**  =0.29 p<0.035 | Sociodemographic, smoking |
|  | C34 (Cancer Bronchus and Lung) | **r (standardized/adjustedR2) increase Ir in CMT**  (Mueller et al. 2015)  =0.31/0.36 p<0.001  **r (standardized/adjustedR2) increase AAMr** **in CMT**  =0.34/0.49 p<0.001 | Sociodemographic, smoking |
|  | C61 (Prostate cancer) | **r increase AAMr in CMT by-tons-of-coal-mined** (Mueller et al. 2015)  *= -0.32 p<0.005: (previously-mined CMT)*  *= -0.27 p<0.047: (recently-mined CMT)* | Sociodemographic, smoking |
|  | Q00, Q01, Q05, Q04.2 (anencephaly, encephalocele, spina bifida, holoprosencephaly) | **RR residents-coal-mining-area/residents-non-coal-mining-area** (Liao et al. 2010)  =1.338(1.004-1.783) p<0.05 | Sociodemographic, smoking, environmental |
|  |  | **Rates of cases in populations < 8km to coal transport roads** (Liao et al. 2016)  = not measure provided (p<0.007) | Environmental |
|  |  | **Mr in villages closer to CM plants vs. control populations** (Gu et al. 2007)  = 281.2/10000, chi-square test (p<0.364) | Gender, environmental,  other comorbidities |

# References

Ahern, M. M., M. Hendryx, J. Conley, E. Fedorko, A. Ducatman, and K. J. Zullig. 2011. 'The association between mountaintop mining and birth defects among live births in central Appalachia, 1996-2003', *Environ Res*, 111: 838-46.

Ahern, Melissa, and Michael Hendryx. 2012. 'Cancer Mortality Rates in Appalachian Mountaintop Coal Mining Areas', *J Environ Occup Sci*, 1: 63-70.

Ahern, Melissa, Martha Mullett, Katherine MacKay, and Candice Hamilton. 2011. 'Residence in Coal-Mining Areas and Low-Birth-Weight Outcomes', *Maternal and Child Health Journal*, 15: 974-79.

Borak, J., C. Salipante-Zaidel, M. D. Slade, and C. A. Fields. 2012. 'Mortality disparities in Appalachia: reassessment of major risk factors', *J Occup Environ Med*, 54: 146-56.

Brink, L. L., E. O. Talbott, S. Stacy, L. P. Marshall, R. K. Sharma, and J. Buchanich. 2014. 'The association of respiratory hospitalization rates in WV counties, total, underground, and surface coal production and sociodemographic covariates', *J Occup Environ Med*, 56: 1179-88.

Buchanich, J. M., L. C. Balmert, A. O. Youk, S. M. Woolley, and E. O. Talbott. 2014. 'General mortality patterns in appalachian coal-mining and non-coal-mining counties', *J Occup Environ Med*, 56: 1169-78.

Christian, W. J., B. Huang, J. Rinehart, and C. Hopenhayn. 2011. 'Exploring geographic variation in lung cancer incidence in Kentucky using a spatial scan statistic: elevated risk in the Appalachian coal-mining region', *Public Health Rep*, 126: 789-96.

Esch, L., and M. Hendryx. 2011. 'Chronic cardiovascular disease mortality in mountaintop mining areas of central Appalachian states', *J Rural Health*, 27: 350-7.

Fernandez-Navarro, P., J. Garcia-Perez, R. Ramis, E. Boldo, and G. Lopez-Abente. 2012. 'Proximity to mining industry and cancer mortality', *Sci Total Environ*, 435-436: 66-73.

Gu, Xue, Liangming Lin, Xiaoying Zheng, Ting Zhang, Xinming Song, Jinfeng Wang, Xinhu Li, Peizhen Li, Gong Chen, and Jilei Wu. 2007. 'High prevalence of NTDs in Shanxi Province: a combined epidemiological approach', *Birth Defects Research Part A: Clinical and Molecular Teratology*, 79: 702-07.

Hendryx, M. 2011. 'Poverty and mortality disparities in central Appalachia: mountaintop mining and environmental justice', *Journal of Health Disparities Research and Practice*, 4: 6.

Hendryx, M. 2009. 'Mortality from heart, respiratory, and kidney disease in coal mining areas of Appalachia', *Int Arch Occup Environ Health*, 82: 243-9.

Hendryx, M., and M. M. Ahern. 2009. 'Mortality in Appalachian coal mining regions: the value of statistical life lost', *Public Health Rep*, 124: 541-50.

Hendryx, M., M. M. Ahern, and T. R. Nurkiewicz. 2007. 'Hospitalization patterns associated with Appalachian coal mining', *J Toxicol Environ Health A*, 70: 2064-70.

Hendryx, M., E. Fedorko, and A. Anesetti-Rothermel. 2010. 'A geographical information system-based analysis of cancer mortality and population exposure to coal mining activities in West Virginia, United States of America', *Geospat Health*, 4: 243-56.

Hendryx, M., E. Fedorko, and J. Halverson. 2010. 'Pollution sources and mortality rates across rural-urban areas in the United States', *J Rural Health*, 26: 383-91.

Hendryx, M., K. O'Donnell, and K. Horn. 2008. 'Lung cancer mortality is elevated in coal-mining areas of Appalachia', *Lung Cancer*, 62: 1-7.

Hendryx, Michael. 2008. 'Mortality rates in Appalachian coal mining counties: 24 years behind the nation', *Environmental Justice*, 1: 5-11.

Hitt, Nathaniel P., and Michael Hendryx. 2010. 'Ecological Integrity of Streams Related to Human Cancer Mortality Rates', *EcoHealth*, 7: 91-104.

Howel, D., T. Pless-Mulloli, and R. Darnell. 2001. 'Consultations of children living near open-cast coal mines', *Environmental Health Perspectives*, 109: 567-71.

Lamm, Steven H., Ji Li, Shayhan A. Robbins, Elisabeth Dissen, Rusan Chen, and Manning Feinleib. 2015. 'Are residents of mountain-top mining counties more likely to have infants with birth defects? The west virginia experience', *Birth Defects Research Part A: Clinical and Molecular Teratology*, 103: 76-84.

Liao, Y., J. Wang, J. Wu, L. Driskell, W. Wang, T. Zhang, G. Xue, and X. Zheng. 2010. 'Spatial analysis of neural tube defects in a rural coal mining area', *Int J Environ Health Res*, 20: 439-50.

Liao, Y., Y. Zhang, L. He, J. Wang, X. Liu, N. Zhang, and B. Xu. 2016. 'Temporal and Spatial Analysis of Neural Tube Defects and Detection of Geographical Factors in Shanxi Province, China', *PLoS ONE*, 11: e0150332.

Liu, A. Y., F. C. Curriero, T. A. Glass, W. F. Stewart, and B. S. Schwartz. 2013. 'The contextual influence of coal abandoned mine lands in communities and type 2 diabetes in Pennsylvania', *Health Place*, 22: 115-22.

Mueller, G. S., A. L. Clayton, W. E. Zahnd, K. M. Hollenbeck, M. E. Barrow, W. D. Jenkins, and D. R. Ruez, Jr. 2015. 'Geospatial analysis of Cancer risk and residential proximity to coal mines in Illinois', *Ecotoxicol Environ Saf*, 120: 155-62.

Pless-Mulloli, T., D. Howel, A. King, I. Stone, J. Merefield, J. Bessell, and R. Darnell. 2000. 'Living near opencast coal mining sites and children's respiratory health', *Occup Environ Med*, 57: 145-51.

Talbott, E. O., R. K. Sharma, J. Buchanich, and S. L. Stacy. 2015. 'Is there an association of circulatory hospitalizations independent of mining employment in coal-mining and non-coal-mining counties in west virginia?', *J Occup Environ Med*, 57: e30-6.

Woolley, S. M., S. L. Meacham, L. C. Balmert, E. O. Talbott, and J. M. Buchanich. 2015. 'Comparison of Mortality Disparities in Central Appalachian Coal- and Non-Coal-Mining Counties', *J Occup Environ Med*, 57: 687-94.
